# Effects of multi-ingredient protein supplementation combined with exercise intervention on body composition and muscle fitness in healthy women: a systematic review with multilevel meta-analysis

**DOI:** 10.3389/fnut.2025.1678433

**Published:** 2025-11-03

**Authors:** Chengyu Zhou, Muen Qiu, Zhuo Zeng, Qi Xie, Kai Xu, Henghao Yan, Bo Wang, Bopeng Qiu, Guoxin Shi

**Affiliations:** ^1^School of Strength and Conditioning, Beijing Sport University, Beijing, China; ^2^School of Medical and Health Sciences, Centre for Human Performance, Edith Cowan University, Joondalup, WA, Australia; ^3^School of Athletic Performance, Shanghai University of Sport, Shanghai, China; ^4^School of Physical Education, Southwest University, Chongqing, China; ^5^Physical Education College, Jilin University, Changchun, China; ^6^Department of Physical Education, Nanjing University of Finance & Economics, Hongshan College, Nanjing, China

**Keywords:** multi-ingredient protein supplements, exercise training, body composition, muscle strength, women, sports nutrition

## Abstract

**Objectives:**

This meta-analysis investigated whether multi-ingredient protein supplements (MIPS) combined with exercise improve body composition and muscle fitness in women. It also examined how participant characteristics, training protocols, and supplementation strategies might influence these outcomes.

**Methods:**

A systematic search of five electronic databases was conducted through February 2025 to identify randomized controlled trials evaluating the effects of MIPS combined with exercise training on body composition and muscle-related outcomes in women. A multilevel meta-analysis was performed to pool effect sizes, reported as standardized mean differences (Hedges’ g), with heterogeneity assessed through predefined subgroup analyses.

**Results:**

Nine randomized controlled trials involving 408 healthy women aged 18 to 73 years were included. The meta-analysis showed that combining MIPS with exercise training led to significant increases in fat-free mass [0.45 kg (0.19 to 0.71), *p* = 0.003], muscle hypertrophy [Hedges’ g = 0.35 (0.05 to 0.65), *p* = 0.027], and muscle strength [Hedges’ g = 0.50 (0.06 to 0.95), *p* = 0.029]. However, no significant effects were observed on fat mass, body fat percentage, waist circumference, or functional performance (all *p* > 0.05). Subgroup analyses revealed that gains in fat-free mass were more pronounced among older adults, overweight individuals, participants whose supplement intake was timed near exercise, and those in interventions exceeding 12 weeks (all *p* < 0.01). Similarly, improvements in muscle hypertrophy and strength were greater in longer interventions and when supplementation was aligned with dietary intake. Younger women showed larger gains in muscle strength, whereas older women experienced more increases in fat-free mass.

**Conclusion:**

Combining MIPS with exercise training significantly improves fat-free mass, muscle mass, and strength in women, with no additional benefits for fat-related or functional outcomes. These effects are moderated by age, BMI, supplementation timing, isocaloric designs, and intervention duration, highlighting the importance of individualized strategies. Further high-quality isocaloric design trials in diverse female populations are needed to refine tailored approaches that optimize health and performance.

**Systematic review registration:**

https://osf.io/hkt7p.

## Graphical abstract

The role of age in ocular toxoplasmosis.
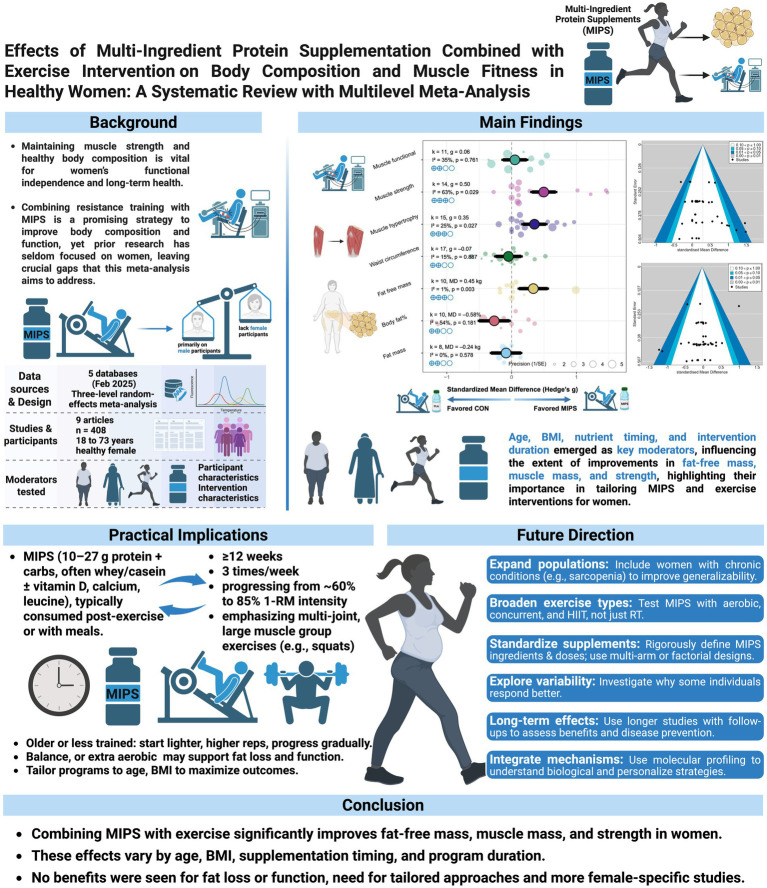

## 1 Introduction

Maintaining muscle strength and a healthy body composition, characterized by sufficient lean mass and a balanced fat distribution, is crucial for overall health and functional independence (1–3). Skeletal muscle facilitates movement and daily activities while also regulating glucose metabolism, supporting basal metabolic rate, and modulating inflammatory processes (4–7). Robust muscle strength helps prevent falls and fractures, delays age-related functional decline, and preserves autonomy in later life (1). Furthermore, maintaining muscle mass reduces visceral fat accumulation and improves fat distribution, thereby decreasing the risk of cardiovascular disease (8), type 2 diabetes, and certain cancers (9). Thus, adequate muscle fitness underpins not only physical performance but also long-term health, enhanced quality of life, and increased longevity. As a result, nutritional interventions, particularly protein supplementation, have gained increasing attention for their potential to improve muscle fitness and body composition, especially when combined with exercise training (10).

Women make up nearly half of the global population, approximately 3.95 billion individuals, and play vital roles in health and social care sectors worldwide (11). At the same time, they experience a marked and progressive decline in physical activity and sports participation from adolescence into older adulthood (12). This trend is largely driven by educational pressures, work commitments, family responsibilities, and increasingly sedentary lifestyles (13). Physical inactivity is strongly associated with reduced muscle strength, unfavorable shifts in decreased lean mass and increased fat accumulation (8), and a detrimental cycle of functional decline that impairs daily activities, accelerates musculoskeletal deterioration, and increases mortality risk (1). Women exhibit sex-specific physiological characteristics, including lower baseline skeletal muscle mass, hormonal fluctuations, and unique patterns of protein metabolism (11). Despite these physiological distinctions, women remain underrepresented in exercise science research, underscoring the need for more tailored and inclusive interventions (14). To address these issues, there is a growing consensus on the value of integrated approaches that combine structured exercise interventions with evidence-based nutritional strategies such as multi-ingredient protein supplements (MIPS) (1–3).

Resistance training (RT), which involves muscle contractions against external loads, has emerged as a highly effective modality for enhancing musculoskeletal health and overall well-being in women As a result, nutritional interventions, particularly protein supplementation, have gained increasing attention for their potential to improve muscle fitness and body composition, especially when combined with exercise training (1). The benefits of RT are further amplified by adequate dietary protein intake, which stimulates muscle protein synthesis and counteracts inactivity or age-related muscle loss (15, 16). Moreover, MIPS, comprising essential amino acids, creatine, vitamin D, and other bioactive compounds, has demonstrated potential to augment the adaptive response to RT. (17) The potential synergistic effects of MIPS and RT may involve several physiological pathways. Essential amino acids, particularly leucine, are thought to stimulate the mechanistic target of rapamycin (mTOR) signaling pathway, which plays a central role in regulating muscle protein synthesis (18). Creatine may support greater training volume and recovery by enhancing phosphocreatine resynthesis (19), while vitamin D is implicated in muscle function through its effects on calcium homeostasis, neuromuscular coordination, and anabolic hormone activity (20). Thus, integrating RT with targeted nutritional supplementation might represent a practical and efficacious strategy to improve body composition and physical function across the female lifespan.

Recent systematic reviews have shown that MIPS combined with exercise can improve muscle strength, lean mass, and body composition, while women remain substantially underrepresented in this literature, comprising only 0–22% of study samples across meta-analyses (17, 21, 22). Given the known sex-based differences in hormonal profiles, protein metabolism, and training responses, extrapolating findings from male cohorts to women is methodologically inappropriate (14), reinforcing the need for female-specific syntheses to guide tailored interventions and summary (23). Additionally, prior studies have predominantly focused on resistance training and middle-aged or older adults, often neglecting the combined effects of different exercise types (e.g., aerobic training) across various age groups in women. Many investigations have also failed to evaluate key indicators of physical function, such as chair stand tests, thereby limiting our understanding of how MIPS affects functional capacity in real-world settings. Collectively, these gaps underscore the need for generating rigorous, female-specific data to support practical exercise and nutritional programming throughout the female lifespan. Lastly, methodologically, prior meta-analyses have often aggregated multiple outcomes from the same study and relied on two-level models that assume effect independence, undermining the precision of pooled estimates (24). To address this, the present study will employ a multilevel meta-analytic framework with robust variance estimation (25), along with moderator analyses to assess the influence of participant characteristics, training modalities, and supplementation protocols.

To address these gaps, the present review aims to systematically evaluate the effects of MIPS combined with exercise training on body composition and functional outcomes in women across different age groups. It will also investigate the moderating roles of participant characteristics, exercise protocols, and supplementation strategies. By synthesizing current evidence, this review seeks to guide precise and individualized interventions that may help preserve muscle health, reduce the risk of metabolic and skeletal disorders, slow functional decline, and ultimately enhance quality of life and longevity in women.

## 2 Methods

This review was conducted in accordance with the Preferred Reporting Items for Systematic Reviews and Meta-Analyses (PRISMA) guidelines (26). The completed PRISMA 2020 checklist is available in Appendix A. This review was prospectively registered in the Open Science Framework (OSF) database^1^ with the identifier https://osf.io/hkt7p.

### 2.1 Literature search

A systematic literature search was conducted across multiple electronic databases, including the Web of Science (Core Collection), PubMed, Cochrane Library, Embase, and Scopus, up to February 21, 2025. The search strategy combined keywords and Medical Subject Headings (MeSH). The search equation was: ((“Multi*” OR “Protein*” OR “Beef Protein” OR “Soy Protein” OR “Pea Protein” OR “Rice Protein” OR “Whey*” OR “BCAA*” OR “Branch*”) AND (“Supplement*” OR “Enrich*” OR “Formula*” OR “Fortifi*”) AND (“Resist*” OR “Endur*” OR “Aerobic Training” OR “Anaerobic Training” OR “Train*” OR “Exercise*” OR “Strength*” OR “Power*” OR “Recov*” OR “Energ*” OR “Performance*”)). Additionally, the reference lists of relevant meta-analyses and original studies were manually reviewed to identify any additional eligible articles.

### 2.2 Study selection

This study utilized EndNote X9 [Clarivate Analytics, 2018] to deduplicate the literature. Subsequently, CZ and MQ independently reviewed the titles and abstracts of the literature according to predefined inclusion and exclusion criteria. In case of discrepancies during the review process, the researchers would convene a meeting to discuss the issues about the established criteria to reach a consensus. If a consensus still could not be reached, a third researcher (GS) would be invited to participate, ultimately deciding whether the literature met the inclusion criteria. In the full text review phase, CZ and MQ also proceeded independently, and the same method used in the title and abstract screening stage was applied to address any discrepancies that arose.

### 2.3 Eligibility criteria

Eligibility criteria were defined *a priori* according to the PICOS framework. Studies were included if they investigated women of any age who were healthy or did not have severe chronic conditions known to substantially limit exercise capacity, such as advanced cardiovascular disease or uncontrolled diabetes. Studies focusing on older women or female athletes were also eligible, provided they met the intervention criteria. Interventions were required to include MIPS administered in combination with structured exercise training (resistance exercise, aerobic exercise, or combined modalities) for a minimum duration of ≥2 weeks. Eligible trials had to report essential elements of both the nutritional and exercise prescriptions, including supplement type and composition, intake frequency, dosage, as well as exercise modality, intensity, frequency, and duration. Studies exclusively examining protein-only supplementation without additional ingredients or lacking a structured exercise component were excluded. Eligible studies included a comparator arm/group involving placebo combined with exercise, exercise without supplementation, or supplementation without exercise. We recognize that comparator conditions varied across trials, which may have contributed to heterogeneity and were considered when interpreting the findings. Primary outcomes included changes in body composition (e.g., lean mass, fat mass, body fat percentage), muscle function (e.g., sit-to-stand performance), muscle strength (e.g., one-repetition maximum, isokinetic testing), and muscle hypertrophy (defined as increases in muscle mass or muscle cross-sectional area). Only randomized controlled trials (RCTs), whether employing parallel-group or crossover designs, were considered.

The following studies were excluded: conference abstracts, editorials, letters, or commentaries; publications in languages other than English or Chinese; and studies involving animal experiments.

### 2.4 Data extraction

Data from all eligible studies were independently extracted by CZ and QX using a standardized, pre-designed form. A third reviewer (HY) cross-checked the extracted information to ensure accuracy and resolve discrepancies. Extracted data included: first author’s name, publication year, participant characteristics (e.g., age, sex, training status), study design (parallel or crossover RCT), details of the intervention and comparator (including supplement formulation, dosage, intake frequency, duration, and exercise prescription), as well as outcome measures and assessment methods.

Specifically, for each eligible group, the following information was recorded: pre- and post-intervention means and standard deviations (SDs), along with sample sizes, for all primary outcomes, including body composition (e.g., lean mass, fat mass, body fat percentage), physical function (e.g., sit-to-stand performance, gait speed, handgrip strength), and muscle strength (e.g., one-repetition maximum, isokinetic testing). When necessary, data were extracted from figures using WebPlotDigitizer or estimated from reported statistics (e.g., confidence intervals, standard errors, or *p*-values) following Cochrane Handbook guidelines. If multiple eligible outcomes were reported within a single study, all relevant data were retained for multilevel analysis. Authors were contacted when essential data were missing or unclear.

This study extracted the mean, standard deviation, and sample size reported for each group both before and after the intervention. We pooled effects using pre- and post-intervention differences (*M* ± SD) for each outcome indicator. The first step is to calculate the difference in means (raw mean difference between post and preintervention for each intervention group):


(1) $$MD_{\mathrm{dif}}f=M_{\text{post}}-M_{\mathrm{pre}}$$

As shown in Equation (1), where ${\mathrm{MD}}_{\text{diff}}\;$ the raw mean difference, $M_{\text{post}}$ is the reported mean post-intervention, and $M_{\mathrm{pre}}$ is the reported mean pre-intervention.

If the study only reported confidence intervals, they were converted to SD using the following formula:


(2) $$\mathrm{SD}=\sqrt{N}\;\frac{CI_{h\mathrm{ig}h}-CI_{\mathrm{low}}}{2t}$$

As shown in Equation (2), where SD is the standard deviation, N is the group sample size, ${\mathrm{CI}}_{\text{high}}\;$ is the upper limit of the confidence interval, ${\mathrm{CI}}_{\mathrm{low}}$ is the lower limit of the confidence interval, and t is the t distribution with N − 1 degrees of freedom the respective confidence level (27).

The SD of the difference in means ( ${\mathrm{SD}}_{\text{diff}}$ ) is calculated as follows (27):


(3) $$SD_{\text{diff}}=\sqrt{S{D_{\mathrm{pre}}}^{2}+S{D_{\text{post}}}^{2}-2r\times SD_{\mathrm{pre}}\times SD_{\text{post}}}$$

As shown in Equations (3, 4), where ${\mathrm{SD}}_{\text{diff}}\;$ is the standard deviation of the difference in means, ${\mathrm{SD}}_{\mathrm{pre}}$ is the standard deviation from pre-intervention, and ${\mathrm{SD}}_{\text{post}}\;$ is the standard deviation from post-intervention. As the original studies included in the meta-analysis did not report Pearson’s correlation coefficients (r) for pre- and post-intervention outcomes, we adopted a correlation coefficient of *r* = 0.5, as recommended by the Cochrane Handbook, which is considered a conservative estimate (27).


(4) $$r=\frac{S{D_{\mathrm{pre}}}^{2}+S{D_{\text{post}}}^{2}-S{D_{ch\text{ange}}}^{2}}{2\times SD_{\mathrm{pre}}\times SD_{\text{post}}}$$

### 2.5 Risk of bias and quality of methods assessment

The risk of bias was assessed independently by CZ and QX using the Cochrane Collaboration’s Risk of Bias tool 2 (RoB 2) (28). This tool evaluates bias across multiple domains, including random sequence generation, allocation concealment, blinding of participants and personnel, blinding of outcome assessment, incomplete outcome data, selective outcome reporting, and other sources of bias. Disagreements between reviewers were resolved through discussion whenever possible. If consensus could not be reached, a third independent reviewer (HY) was consulted to adjudicate. Additionally, the Physiotherapy Evidence Database (PEDro) scale was used to assess the methodological quality of included studies (29). The PEDro scale rates studies on a scale from 0 to 10, with scores of ≥6 indicating high quality, scores of 4–5 indicating moderate quality, and scores ≤3 considered low quality.

### 2.6 Statistical analysis

All statistical analyses were performed using R (version 4.2.1) (30). Given that several included studies reported multiple experimental groups or outcomes, a traditional two-level meta-analysis could violate the assumption of independence and potentially inflate precision due to duplicated data structures (24). To address this, we implemented a three-level random-effects meta-analysis, following the framework proposed by Assink and Wibbelink (31). This model decomposes total variance into three levels: Level 1 (sampling variance), Level 2 (within-study variance), and Level 3 (between-study variance), thereby accommodating dependency among effect sizes and the hierarchical data structure (32). To further account for statistical dependencies, a cluster-robust variance estimation (CRVE) approach based on a variance–covariance matrix was applied, along with small-sample adjustments to ensure unbiased standard errors in the presence of correlated outcomes (25). Retaining all available effect sizes from each study, rather than averaging or discarding them, enhanced statistical power and improved estimation precision (31). A random-effects modeling approach was adopted to account for expected heterogeneity in study design, intervention protocols, and participant populations. Model parameters were estimated using restricted maximum likelihood (REML), and results were cross-validated using maximum likelihood (ML) estimation to ensure robustness (33).

Effect sizes were calculated using either mean difference (MD) or standardized mean difference (SMD), based on the homogeneity of outcome units across studies. Following Cochrane Handbook recommendations, MDs were used for outcomes measured on a consistent scale (e.g., kilograms or percentages), while SMDs were applied when studies used different scales or instruments for the same construct. Specifically, the following outcomes were analyzed using MD: fat mass, fat mass percentage, and fat-free mass. All other outcomes, including body composition and muscle fitness indicators, were synthesized using SMD. Hedges’ g was chosen as the standardized metric to correct for small sample bias. The magnitude of g was interpreted as trivial (<0.2), small (0.2–0.5), moderate (0.5–0.8), and large (>0.8) (34).

To assess between-study heterogeneity, we reported Cochrane’s Q, the *I*^2^ statistic, *τ*^2^, and τ values, along with 95% confidence intervals (CIs) and 95% prediction intervals (PIs) to represent the dispersion of true effects across studies (35). As widely recommended in current methodological literature, *I*^2^ was used as the primary index for heterogeneity. The values of I^2^ were interpreted as follows: 0–25% (low heterogeneity), 25–50% (moderate), 50–75% (substantial), and 75–100% (considerable) (27). To evaluate the statistical power of the pooled effect estimates and minimize type II error, power analyses were conducted using the “*metameta*” package (36).

Furthermore, subgroup analyses were conducted to examine potential moderators and to explore sources of heterogeneity across both categorical and continuous variables (37). These analyses focused on three primary domains: participant characteristics (age group and BMI classification), intervention characteristics (nutrient timing and intervention duration), and training protocol parameters. Based on theoretical rationale and the availability of data, four key subgroup variables were selected for detailed exploration: (1) age group (<65 vs. ≥65 years), (2) BMI classification (<25 kg/m^2^, 25.0–29.9 kg/m^2^, and ≥30 kg/m^2^), (3) nutrient timing (near training vs. near dietary intake), (4) protein dosage [<0.25 vs. ≥0.25 g/kg/day (38)], (5) caloric equivalence of study design (isocaloric vs. non-isocaloric RCTs), and (6) intervention duration (≤12 weeks vs. >12 weeks).

Finally, sensitivity analyses were performed using leave-one-out methods to identify any influential studies that might have affected the overall results. Publication bias was assessed using contour-enhanced funnel plots and Egger’s regression test, with a *p*-value greater than 0.05 considered indicative of no significant publication bias (39).

### 2.7 Certainty of the evidence

Evidence of effectiveness for each study was combined with quality scores for use in discussing the results. The Grading of Recommendations Assessment, Development, and Evaluation (GRADE) methodology was used to rate the certainty of the evidence as “high,” “moderate,” “low,” or “very low” (40). GRADE was completed by two researchers, with differences resolved through consensus. This comprehensive assessment rates evidence as follows: (1) the risk of bias, downgraded by one level if “some concerns” and two levels if “high risk” of bias; (2) inconsistency, downgraded by one level when the impact of statistical heterogeneity (*I*^2^) is moderate (>25%) and by two levels when high >75%; (3) imprecision: downgraded by one level when statistical power < 80% and if there was no clear direction of the effects (41); (4) risk of publication bias: downgrade one level if Egger’s test < 0.05.

## 3 Results

### 3.1 Studies retrieved

**Figure 1:**
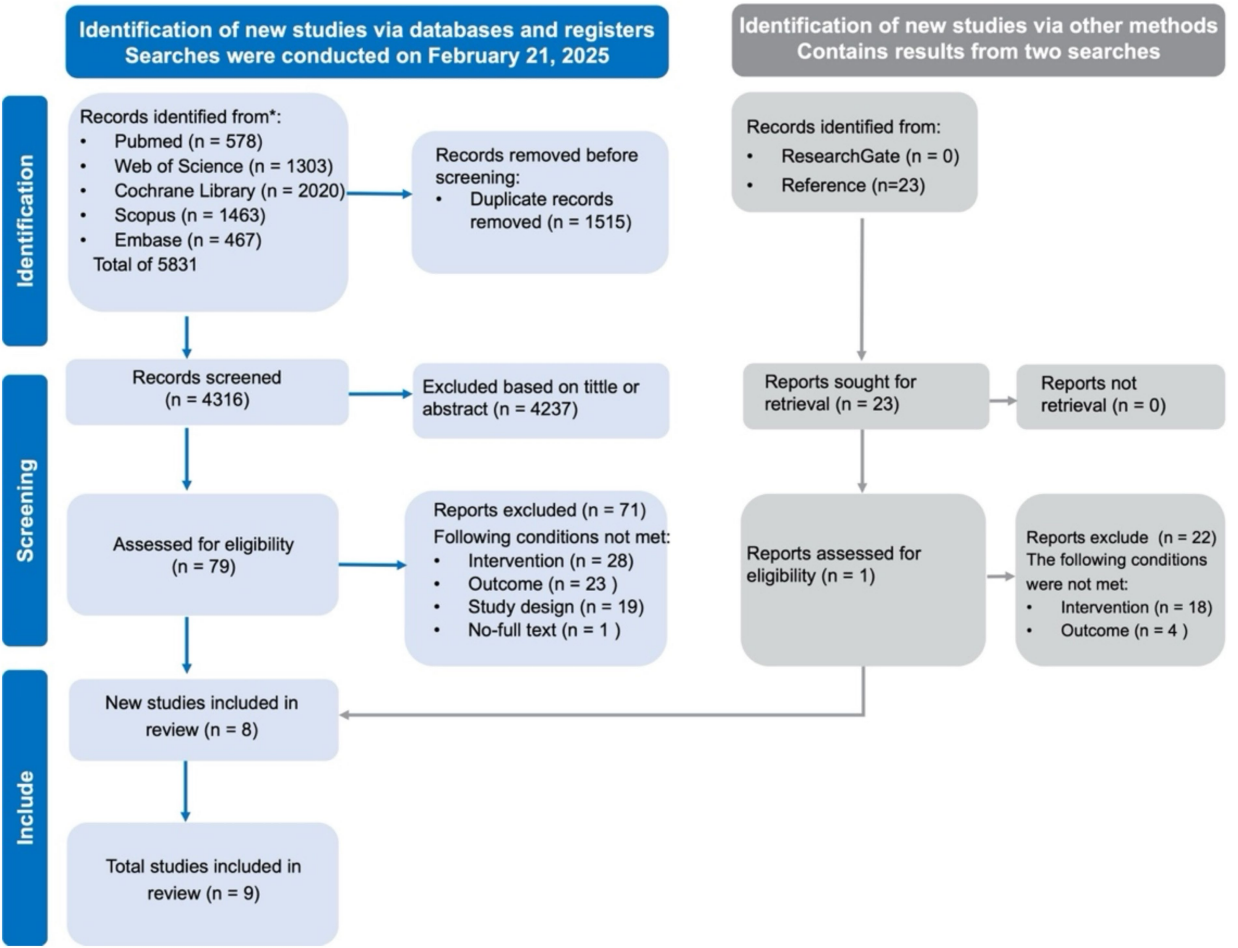
PRISMA flow diagram of study selection.

A systematic search across five databases, along with additional reference checks, initially identified 5,831 records. After removing duplicates and screening titles and abstracts, 79 articles underwent full-text review, nine studies met the inclusion criteria and were included in this review (42–50). The detailed selection process is shown in Figure 1.

### 3.2 Characteristics of included studies

All nine included studies were randomized controlled trials (RCTs) involving healthy women without clinically diagnosed chronic diseases (42–50). A total of 408 participants were enrolled, with ages ranging from approximately 18 to 73 years and BMI values typically between 21.1 and 36.5 kg/m^2^. All studies incorporated structured exercise training combined with MIPS. RT was the most frequently employed exercise modality, followed by aerobic training and combined RT plus aerobic training. Intervention durations ranged from 4 to 24 weeks, with most studies prescribing training two to three times per week. Exercise intensity typically ranged from 60 to 85% of one-repetition maximum (1-RM) for RT, or 40 to 65% of heart rate reserve for aerobic protocols. Regarding supplementation strategies, the most common MIPS formulations combined protein with carbohydrates, followed by those including vitamin D and/or calcium. Less frequently, supplements contained leucine, other essential amino acids, or creatine. Most interventions administered supplements post-exercise or with meals. Among the nine included RCTs, protein sources comprised animal-based (red meat, 1 study) (46), plant-based (soy protein, 2 studies) (42, 50), and predominantly dairy-based proteins (6 studies) (43–45, 47–49). The protein dosages administered across trials varied considerably, ranging from 10 g/day (≈0.15 g/kg/day) to 45 g/day (≈0.6 g/kg/day). Among the included trials, four were designed as isocaloric RCTs (42, 47–49) and five as non-isocaloric RCTs (43–46, 50). The majority of studies reported detailed information on supplement dosage, frequency, macronutrient composition, and caloric content, enabling a comprehensive characterization of intervention protocols. Detailed participant and protocol characteristics for each study are summarized in Table 1.

**Table 1:** The characteristics of the studies included.

Author	Group	*N*	Age	BMI	MIPS/supplement protocol	Exercise protocol	Fre	Week	PEDro	Design	Main finding
Daly et al. (46)	MIPS+RT	53	72.1	27.7	Type: Lean red meat and vitamin D₃ (Animal-based) Details: Two 80-g lean meat cooked/day (~160 g/day) + 1,000 IU vitamin D₃/day Protein dosage: 45 g, 0.6/kg/day Timing: After RT	Type: RT Mode: Free weights Intensity: RPE 14–16 Volume: 3 sets × 8–12 reps	2	16	7	Non-isocaloric RCT	Compared with RT, MIPS+RT showed greater increases in lean tissue mass, leg muscle mass and strength, ↑ insulin-like growth factor I, and ↓ interleukin-6; no adverse effects (lipids/blood pressure).
	RT	48	73.6	27.6	Type: Placebo (Carbohydrate and vitamin D₃) Details: 72 kcal, 11.6 g protein, 0.4 g fat, 5.5 g carbohydrate, 87 mg sodium Timing: After AT	Type: RT Mode: Free weights Intensity: RPE 14–16 Volume: 3 sets × 8–12 reps	2	16			
Li et al. (50)	MIPS+AT	8	38	21.11	Type: Soy protein (Plant-based) Details: Daily consumption provided 72 kilocalories, with 11.6 grams of protein, 0.4 grams of fat, 5.5 grams of carbohydrates, a of sodium. Protein dosage: 23 g, 0.4/kg/day Timing: After AT	Type: AT Mode: Fighting action Intensity: 40–65% HR_R_ Volume: 60 min/session	2	8	6	Non-isocaloric RCT	Compared with the AT group, the AT + MIPS group had significant reductions in body weight, body mass index, body fat percentage, girth (waist circumference and hip circumference), and lean body mass percentage
	AT	8	34	23.55	Type: Placebo (water) Details: Equal volume of water Timing: After AT	Type: AT Mode: Fighting action Intensity: 40–65% HR_R_ Volume: 60 min/session	2	8			
Maesta et al. (42)	MIPS+RT	14	57.6	27.8	Type: Soy protein (Plant-based) Details: Each 25 g of soy protein added to a glass of skimmed milk contained 0.31 g of lipids, 12.2 g proteins, 10 g carbohydrates, 0.7 g fiber, and 92 kcal. Protein dosage: 12 g, 0.2/kg/day Timing: Breakfast or lunch	Type: RT Mode: whole-body program Intensity: 60–80% of 1-RM Volume: 3 sets × 8–12 reps	3	16	6	Isocaloric RCT	Muscle mass in both the MIPS+RT group and the RT group increased significantly, and waist circumference decreased. Among the population that consumed MIPS alone, the average values of total cholesterol and Low-Density Lipoprotein cholesterol were significantly reduced.
	RT	11	60.7	27.7	Type: Placebo (isocaloric maltodextrin) Details: 25 g of maltodextrin every day Timing: Breakfast or lunch	Type: RT Mode: whole-body program Intensity: 60–80% of 1-RM Volume: 3 sets × 8–12 reps	3	16			
	MIPS	10	61.3	27.2	Type: Soy protein (Plant-based) Details: Each 25 g of soy protein added to a glass of skimmed milk contained 0.31 g of lipids, 12.2 g proteins, 10 g carbohydrates, 0.7 g fiber, and 92 kcal. Protein dosage: 12 g, 0.2/kg/day Timing: Breakfast or lunch	No training	n/a	16			
	CON	11	57.9	26.6	Type: Placebo (isocaloric maltodextrin) Details: 25 g of maltodextrin every day Timing: Breakfast or lunch	No training	n/a	16			
Ormsbee et al. (47)	MIPS+CT-1	13	27.7	33.1	Type: Whey protein (Dairy-based) Details: Each 38 g serving contained either (a) 30 g whey protein (50% isolate + 50% concentrate), 4 g carbohydrate, 1.5 g fat, 150 kcal Protein dosage: 30 g/day, ≈0.32 g/kg/day Timing: After dinner	Type: RT + HIIT Mode: chest press and leg press Intensity: 70–85% of 1RM Volume: 3 sets × 10 reps	3	4	6	Isocaloric RCT	The morning satiety in the MIPS+CT-2 group was significantly higher than that in the MIPS+CT-1 or RT groups. Exercise training increased lean body mass and strength in all groups, reduced body fat, and improved emotional state.
	MIPS+CT-2	14	29.3	34.4	Type: Casein protein (Dairy-based) Details: 30 g micellar casein protein, 3 g carbohydrate, 0.5 g fat, 140 kcal Protein dosage: 30 g/day, ≈0.32 g/kg/day Timing: After dinner	Type: RT + HIIT Mode: chest press and leg press Intensity: 70–85% of 1RM Volume: 3 sets × 10 reps	3	4			
	CT	10	30	36.5	Type: Placebo (Maltodextrin and fat) Details: Take 34 g of maltodextrin and 2 g of fat every day Timing: After dinner	Type: RT + HIIT Mode: chest press and leg press Intensity: 70–85% of 1RM Volume: 3 sets × 10 reps	3	4			
Holm et al. (43)	MIPS+RT	13	55	24	Type: Whey protein (Dairy-based) Details: Each serving contained 10 g whey protein, 31 g carbohydrate, 1 g fat, 5.0 μg vitamin D, 250 mg calcium, total energy 730 kJ. The placebo contained 6 g carbohydrate and 12 mg calcium, total energy 102 kJ. Protein dosage: 10 g/day, ≈0.15 g/kg/day Timing: After RT	Type: RT Mode: Supine leg press (high-foot and low-foot positions), knee extension, sit-ups, back extensions, latissimus pull-down Intensity: Low- Moderate Volume: 3 sets × 15 reps	3	24	6	Non-isocaloric RCT	Compared with RT, the centripetal force and isokinotropic muscle strength in the MIPS group increased, and the lean body mass decreased significantly. The lumbar bone mineral density responses of the two groups of patients were similar, but the improvement degree of femoral neck bone mineral density was greater in the nutritional group
	RT	16	55	27	Type: Placebo (Carbohydrates and calcium) Details: Consume 6 g of 102 kJ of carbohydrates and 12 mg of calcium every day Timing: After RT	Type: RT Mode: Supine leg press (high-foot and low-foot positions), knee extension, sit-ups, back extensions, latissimus pull-down Intensity: Low- Moderate Volume: 3 sets × 15 reps	3	24			
Nabuco et al. (48)	MIPS+RT-1	22	67.5	26.4	Type: Whey protein (Dairy-based) Details: Each 200 mL serving contained 27.1 g hydrolyzed whey protein, 5.2 g carbohydrate, 0.2 g fat, total energy 131 kcal. Protein dosage: 27.1 g/day, 0.4 g/kg/day Timing: Either pre-RT or post-RT.	Type: RT Mode: whole-body program Intensity: 65–80% 1RM Volume: 3 sets × 10 reps	3	12	6	Isocaloric RCT	Compared with RT alone, both MIPS+RT-1 and MIPS+RT-2 produced greater improvements in skeletal muscle mass and strength, as well as more pronounced reductions in walking time in the 10-meter walk test.
	MIPS+RT-2	21	66.2	25.3	Type: Whey protein (Dairy-based) Details: Each 200 mL serving contained 27.1 g hydrolyzed whey protein, 5.2 g carbohydrate, 0.2 g fat, total energy 131 kcal. Protein dosage: 27.1 g/day, 0.4 g/kg/day Timing: Either pre-RT or post-RT.	Type: RT Mode: whole-body program Intensity: 65–80% 1RM Volume: 3 sets × 10 reps	3	12			
	RT	23	66.5	23.8	Type: Placebo (protein and carbohydrate) Details: The placebo contained 0.3 g protein and 33.3 g carbohydrate, total energy 134 kcal. Timing: Either pre-RT or post-RT.	Type: RT Mode: whole-body program Intensity: 65–80% 1RM Volume: 3 sets × 10 reps	3	12			
Nabuco et al. (49)	MIPS+RT-1	22	67.5	26.4	Type: Whey protein (Dairy-based) Details: Each 200 mL serving contained 27.1 g hydrolyzed whey protein, 5.2 g carbohydrate, 0.2 g fat, total energy 131 kcal. Protein dosage: 27.1 g/day, 0.4 g/kg/day Timing: Either pre-RT or post-RT.	Type: RT Mode: whole-body program Intensity: 65–80% 1RM Volume: 3 sets × 10 reps	3	12	6	Isocaloric RCT	Compared with RT alone, both MIPS+RT interventions produced greater improvements in muscle mass and lipid profile, while post-RT whey protein intake additionally reduced body fat and improved body composition indices.
	MIPS+RT-2	21	66.2	25.3	Type: Whey protein (Dairy-based) Details: Each 200 mL serving contained 27.1 g hydrolyzed whey protein, 5.2 g carbohydrate, 0.2 g fat, total energy 131 kcal. Protein dosage: 27.1 g/day, 0.4 g/kg/day Timing: Either pre-RT or post-RT.	Type: RT Mode: whole-body program Intensity: 65–80% 1RM Volume: 3 sets × 10 reps	3	12			
	RT	23	66.5	23.8	Type: Placebo (protein and carbohydrate) Details: The placebo contained 0.3 g protein and 33.3 g carbohydrate, total energy 134 kcal. Timing: Either pre-RT or post-RT.	Type: RT Mode: whole-body program Intensity: 65–80% 1RM Volume: 3 sets × 10 reps	3	12			
Leenders et al. (45)	MIPS+RT	12	72	24.2	Type: Milk protein concentrate (Dairy-based) Details: Each 250 mL package contained 15 g milk protein, 0.5 g fat, 7.13 g lactose, and 0.42 g calcium, providing a total of 389 kJ. Protein dosage: 15 g/day, ≈0.24 g/kg/day Timing: After breakfast	Type: RT Mode: leg press and leg extension Intensity: 60–80% 1RM Volume: 3–4 sets × 8–15 reps	3	24	6	Non-isocaloric RCT	Both RT and MIPS+RT significantly enhanced strength, muscle mass, quadriceps cross-sectional area, and functional performance, with no additional benefits observed for MIPS+RT over RT alone.
	RT	12	69	25	Type: Placebo (Lactose and calcium) Details: The placebo contained 7.13 g lactose and 0.42 g calcium, providing 119 kJ, with no protein or fat. Timing: After breakfast	Type: RT Mode: leg press and leg extension Intensity: 60–80% 1RM Volume: 3–4 sets × 8–15 reps	3	24			
White et al. (44)	MIPS+RT	12	18.8	NR	Type: Yogurt (Dairy-based) Details: Each 6-oz (≈170 g) serving of Yoplait yogurt contained 5 g protein, 19 g carbohydrate, 0 g fat, 200 mg calcium, 80 IU vitamin D, and a total energy of 100 kcal. Participants consumed 3 servings per day. Protein dosage: 15 g/day total, ≈0.21 g/kg/day Timing: After training	Type: RT Mode: whole-body program Intensity: 60–80% 1RM Volume: 3–4 sets × 8–15 reps	3	8	5	Non-isocaloric RCT	Calories and protein significantly increased from baseline for MIPS. Fat-free mass increased and body fat% decreased for all groups with training, but Group Time interactions were not observed. Resting metabolic rate and fat oxidation did not change with training for any group.
	RT	11	19.3	NR	Type: Placebo (carbohydrate) Details: Each serving (Clif Shot) contained 25 g carbohydrate, 0 g protein, 0 g fat, and total energy 100 kcal. Timing: After training	Type: RT Mode: whole-body program Intensity: 60–80% 1RM Volume: 3–4 sets × 8–15 reps	3	8			

N, sample size; Age, mean age of participants (years); BMI, body mass index (kg/m^2^); MIPS, multi-ingredient protein supplementation; RT, resistance training; AT, aerobic training; CT, combined training (aerobic + resistance training); HIIT, high-intensity interval training; CON, control; NR, not reported; Fre, training frequency (sessions per week); Week, duration of intervention (weeks); PEDro, Physiotherapy Evidence Database scale (study quality score); Design, study design (e.g., isocaloric RCT, non-isocaloric RCT); RPE, rating of perceived exertion; RM, repetition maximum; HRR, heart rate reserve; HRmax, maximal heart rate; IU, international unit (vitamin D dosage); kJ, kilojoules (energy); kcal, kilocalories (energy); ES, effect size; SD, standard deviation.

### 3.3 Primary analysis

**Figure 2:**
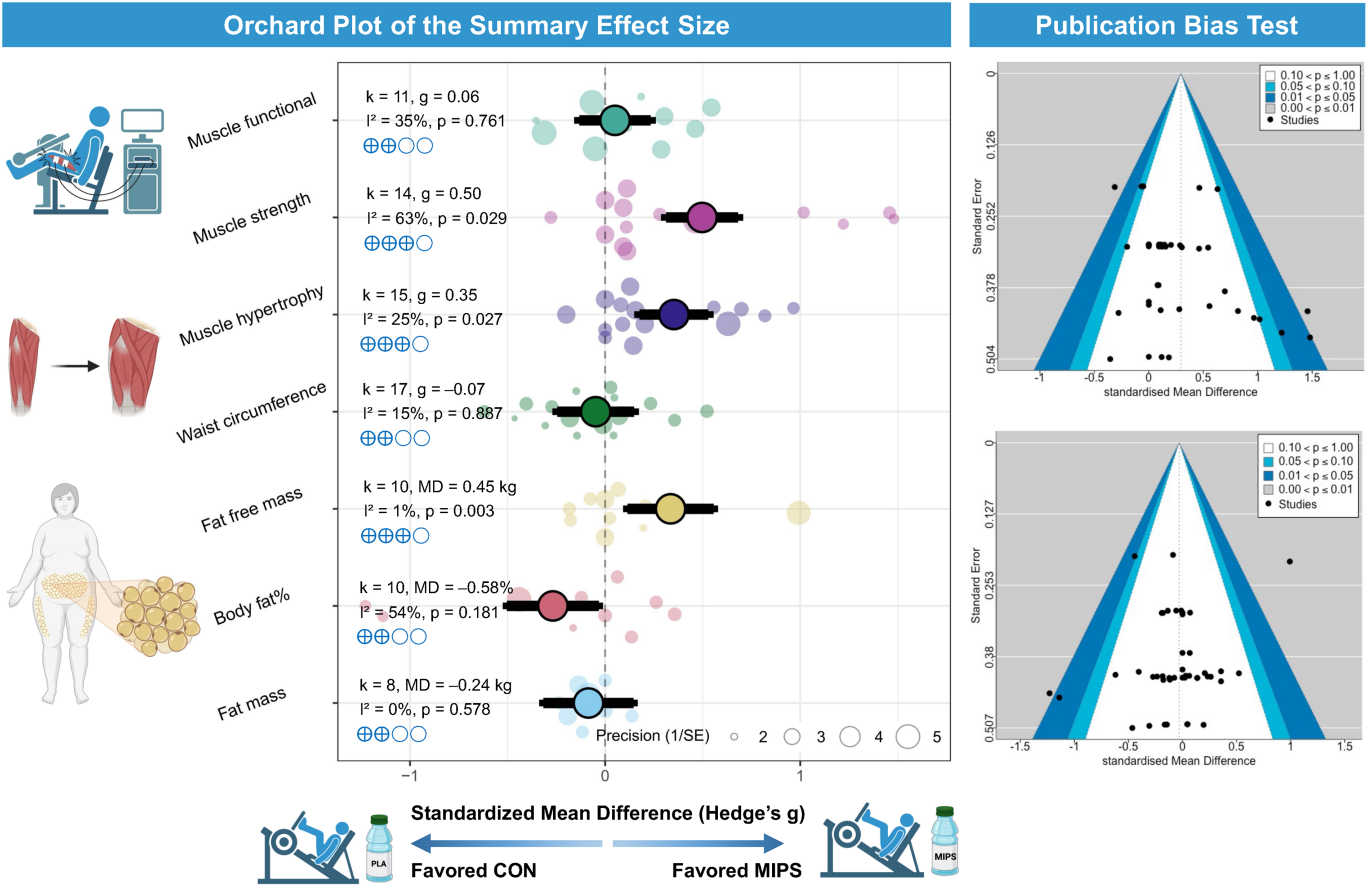
Effects of MIPS combined with exercise training on body composition and muscle-related. Left panel: Orchard plot illustrating the standardized mean differences (Hedges’ g) and 95% confidence intervals for the effects of multi-ingredient protein supplementation (MIPS) combined with exercise training compared to control groups on various outcomes, including muscle functional performance, muscle strength, muscle hypertrophy, waist circumference, fat-free mass, body fat percentage, and fat mass. Larger points and thicker branches represent summary effects for each outcome, while smaller dots indicate individual study effects. Right panel: Funnel plots evaluating publication bias for the primary outcomes. The distribution of studies suggests no substantial asymmetry, indicating a low risk of publication bias.

In terms of body composition, the meta-analysis revealed that combining MIPS with exercise training did not produce statistically significant reductions in fat mass [k = 8, MD = −0.24 kg; 95% CI: (−1.20, 0.73); *I*^2^ = 0%; PI: (−1.20, 0.73); *p* = 0.578; Figure 2], fat mass percentage [*k* = 10, MD = −0.58%; 95% CI: (−1.48, 0.32); *I*^2^ = 54.2%; PI: (−2.39, 1.24); *p* = 0.181; Figure 2], or waist circumference [*k* = 17, g = −0.07; 95% CI: (−1.16, 1.01); *I*^2^ = 15.3%; PI: (−1.70, 1.56); *p* = 0.887; Figure 2]. However, a statistically significant increase in fat-free mass was observed [*k* = 10, MD = 0.45 kg; 95% CI: (0.19, 0.71); *I*^2^ = 1.1%; PI: (0.15, 0.75); *p* = 0.003; Figure 2].

Regarding muscle fitness, MIPS combined with exercise training resulted in significant improvements in muscle hypertrophy [*k* = 15, g = 0.35; 95% CI: (0.05, 0.65); *I*^2^ = 25.2%; PI: (−0.16, 0.86); *p* = 0.027; Figure 2] and muscle strength [*k* = 14, g = 0.50; 95% CI: (0.06, 0.95); *I*^2^ = 63.0%; PI: (−0.54, 1.55); *p* = 0.029; Figure 2]. In contrast, no significant enhancement was found in functional performance [*k* = 11, g = 0.06; 95% CI: (−0.37, 0.49); *I*^2^ = 34.5%; PI: (−0.58, 0.70); *p* = 0.761; Figure 2].

The statistical power of the pooled main effects assessed via sunset plots is presented in Appendix B. Detailed forest plots for all individual outcomes are provided in Appendices C and D.

### 3.4 Secondary analysis

Subgroup analyses were conducted to explore modifying effects on fat mass and body fat (%), and no significant subgroup effects were found for age, BMI classification, intervention timing, protein dosage, caloric equivalence of study design, and intervention duration (all *p* > 0.05).

Subgroup analyses on fat-free mass revealed significant modifying effects of age, BMI category, nutrient timing, caloric equivalence, and intervention duration (all subgroup difference *p* < 0.01). When stratified by age, a significant improvement in fat-free mass was observed among older female adults (0.48 kg), while no significant effect was found in young female adults (−0.22 kg). For BMI, significant gains were observed in overweight female adults (0.47 kg), but not in those female adults with obesity (−0.34 kg). Regarding nutrient timing, fat-free mass significantly increased when supplementation was timed near training sessions (0.48 kg), whereas no significant effect was observed when timed near dietary intake (−0.04 kg) in female adults. Caloric equivalence significantly moderated the results (p for interaction = 0.003): non-isocaloric RCTs showed significant gains in fat-free mass (0.49 kg, *p* = 0.001), whereas isocaloric RCTs demonstrated no effect (−0.16 kg, *p* = 0.68). For intervention duration, significant improvements were found in studies lasting more than 12 weeks (0.49 kg), but not in those with shorter durations (−0.14 kg) in female adults. Additionally, protein dosage did not significantly moderate the effects with no subgroup differences (*p* = 0.45).

Subgroup analyses on muscle mass revealed significant modifying effects of nutrient timing, caloric equivalence, and intervention duration in female adults (all subgroup difference *p* < 0.01). Regarding nutrient timing, muscle mass gains were greater when supplementation was timed near dietary intake (*g* = 0.45) compared to near training (*g* = 0.29) in female adults. For intervention duration, larger improvements were observed in studies lasting more than 12 weeks (*g* = 0.46), whereas shorter interventions showed minimal effects (*g* = 0.07) in female adults. Caloric equivalence emerged as a potential moderator: non-isocaloric RCTs showed significant gains in muscle mass (*g* = 0.46, *p* = 0.008), whereas isocaloric RCTs did not demonstrate a significant effect (*g* = 0.18, *p* = 0.22). Additionally, protein dosage did not significantly moderate the effects with no subgroup differences (*p* > 0.05).

Subgroup analyses on muscle strength revealed significant modifying effects of age, nutrient timing, caloric equivalence, and intervention duration in female adults. When stratified by age, greater improvements were observed in young female adults (*g* = 0.71) compared to older female adults (*g* = 0.41). Regarding nutrient timing, muscle strength gains were greater when supplementation was timed near dietary intake (*g* = 0.76) compared to near training (*g* = 0.44) in female adults. For intervention duration, studies lasting more than 12 weeks showed greater effects (*g* = 0.86) than those with shorter durations (*g* = 0.27) in female adults. Caloric equivalence showed a moderator: non-isocaloric RCTs demonstrated significant improvements in muscle strength (*g* = 0.71, *p* = 0.018), whereas isocaloric RCTs showed smaller, non-significant effects (*g* = 0.32, *p* = 0.31). Additionally, protein dosage did not significantly moderate the effects with no subgroup differences (*p* > 0.05).

### 3.5 Risk of Bias and methodological quality

**Figure 3:**
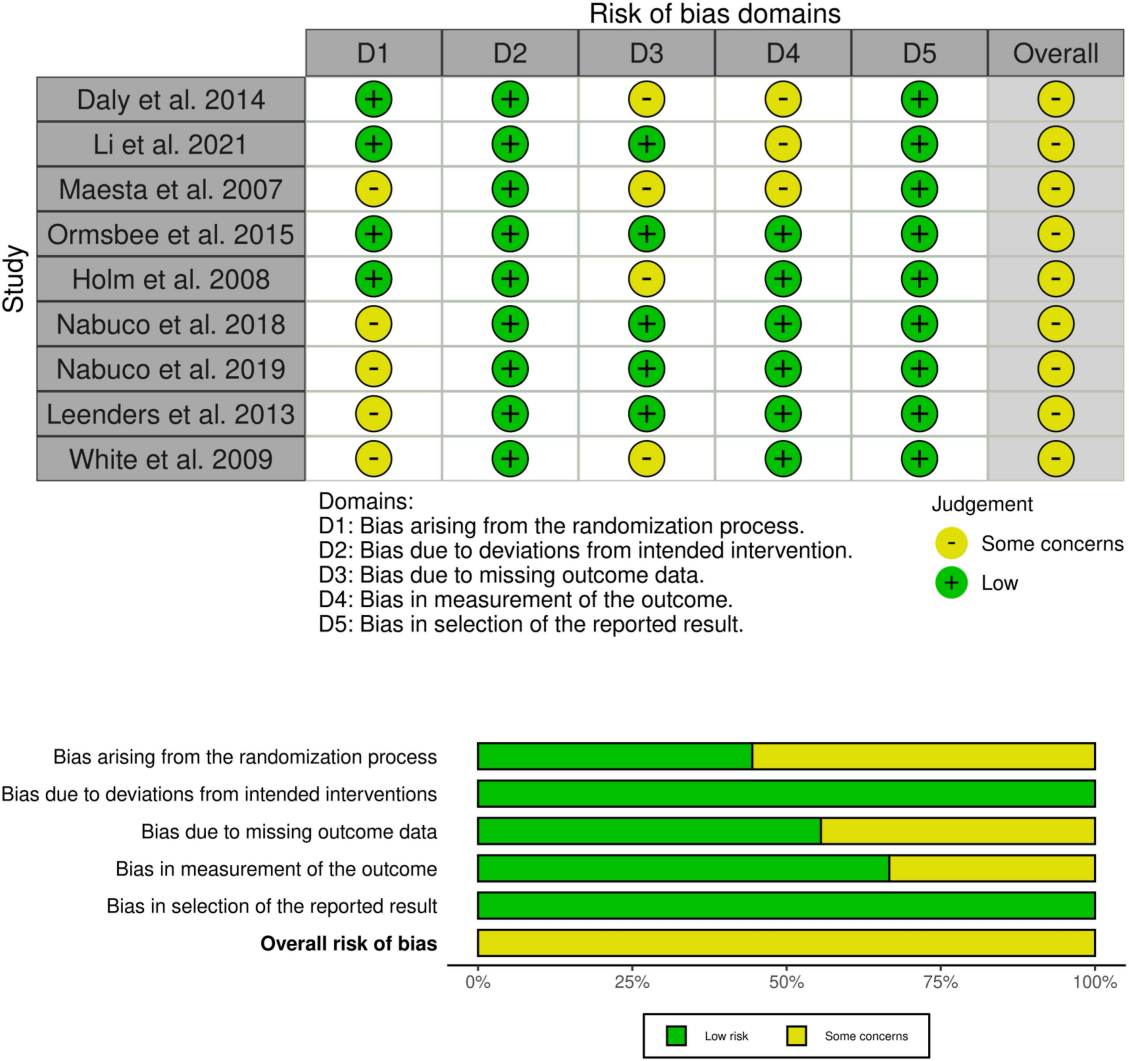
Risk of bias assessment diagram.

Risk of bias was assessed and reported for each included study (Figure 3). Overall, all studies were judged to present “some concerns “regarding risk of bias. Specifically, 55% of studies did not report allocation concealment, thereby presenting some concerns in the randomization process. Additionally, 44% of studies had some degree of participant attrition, leading to concerns related to incomplete outcome data. Furthermore, 33% of studies did not report adequate blinding of outcome assessment, which also contributed to a judgment of some concerns in this domain. Importantly, sensitivity analyses indicated that excluding any study, including those at higher risk of bias, did not alter the main pooled results (see Sensitivity Analyses section).

The PEDro scale (Table 2) assessment indicated that the overall methodological quality of the included studies was predominantly moderate. Most trials adequately defined eligibility criteria, used random allocation, achieved baseline comparability, and provided appropriate statistical analyses with measures of variability. However, key methodological limitations were evident, including the lack of blinding of participants, therapists, and, often, outcome assessors, which is a common challenge in exercise intervention studies. Additionally, intention-to-treat analyses were rarely conducted, introducing a potential source of bias. These factors underscore the need for cautious interpretation of the findings, although the methodological rigor was generally sufficient to support the primary conclusions of this meta-analysis.

**Table 2:** PEDro scale assessment results.

Author, year	D1	D2	D3	D4	D5	D6	D7	D8	D9	D10	D11	Total
Daly et al. (46)	Y	1	1	1	1	0	0	1	0	1	1	7
Holm et al. (43)	Y	1	1	1	1	0	0	0	0	1	1	6
Leenders et al. (45)	Y	1	0	1	1	0	1	0	0	1	1	6
Li et al. (50)	Y	1	0	1	1	0	0	1	0	1	1	6
Maesta et al. (42)	Y	1	0	1	1	0	0	1	0	1	1	6
Nabuco et al. (49)	Y	1	0	1	1	1	0	0	0	1	1	6
Nabuco et al. (48)	Y	1	0	1	1	1	0	0	0	1	1	6
Ormsbee et al. (47)	N	1	0	1	1	1	0	0	0	1	1	6
White et al. (44)	Y	1	0	1	0	0	1	0	0	1	1	5

Studies scoring ≥6 is considered high quality, those scoring 4–5 are considered moderate quality, and those scoring ≤3 are considered low quality. 1. eligibility criteria were specified (not included in the total score). 2. subjects were randomly allocated to groups (in a crossover study, subjects were randomly allocated an order in which treatments were received). 3. allocation was concealed. 4. the groups were similar at baseline regarding the most important prognostic indicators. 5. there was blinding of all subjects. 6. there was blinding of all therapists who administered the therapy. 7. there was blinding of all assessors who measured at least one key outcome. 8. measures of at least one key outcome were obtained from more than 85% of the subjects initially allocated to groups. 9. all subjects for whom outcome measures were available received the treatment or control condition as allocated or, where this was not the case, data for at least one key outcome was analyzed by “intention to treat”. 10. the results of between-group statistical comparisons are reported for at least one key outcome. 11. the study provides both point measures and measures of variability for at least one key outcome.

### 3.6 Sensitivity analysis

Sensitivity analyses indicated that the pooled estimates for all outcomes remained stable, with no substantial changes observed upon the exclusion of any single study. This suggests that the overall results were not unduly influenced by any individual trial. Detailed findings from the sensitivity analyses are provided in Appendices E and F.

### 3.7 Certainty of evidence

**Figure 4:**
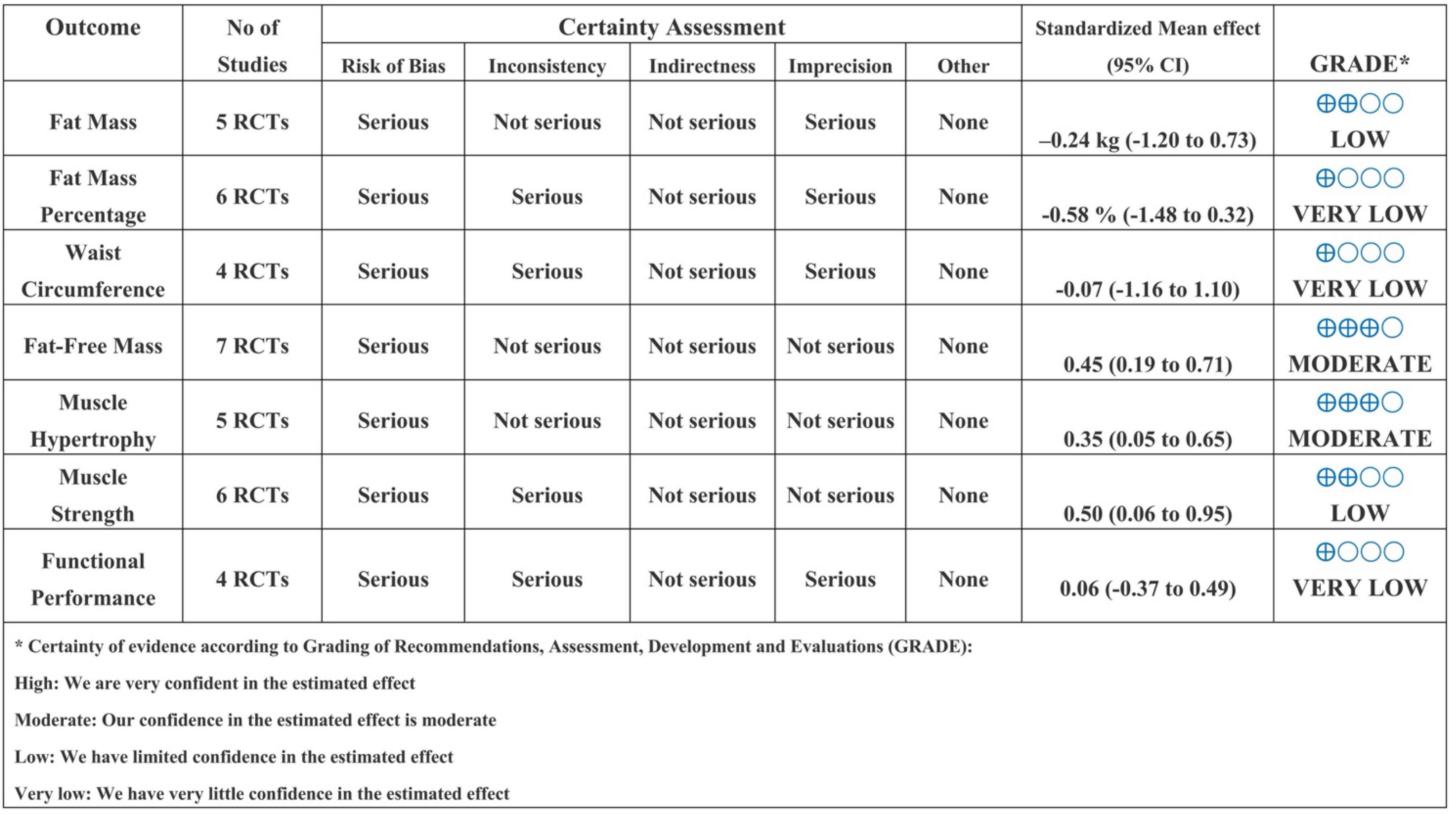
GRADE of all outcomes.

According to the GRADE framework, the certainty of evidence ranged from very low to moderate across outcomes (Figure 4). Specifically, the certainty was rated as very low for waist circumference, fat mass percentage, and functional performance, primarily due to serious concerns regarding risk of bias and inconsistency. The evidence was graded as low certainty for fat mass and muscle strength outcomes, again reflecting limitations associated with study quality and heterogeneity. In contrast, moderate certainty was observed for fat-free mass and muscle hypertrophy, indicating greater confidence in these effect estimates despite some identified concerns. Overall, the body of evidence was most robust for fat-free mass and muscle hypertrophy, whereas conclusions related to changes in fat mass distribution, waist circumference, and functional performance should be interpreted cautiously.

## 4 Discussion

This meta-analysis is the first to systematically assess the effects of MIPS combined with exercise training in women, addressing a critical gap in the current literature. Despite growing awareness of sex-specific differences in exercise science, women remain significantly underrepresented, particularly in studies on protein supplementation. While the findings suggest that such interventions might improve fat-free mass, muscle mass, and muscle strength, they should be interpreted with caution due to the limited number of included trials (n = 9) and relatively small sample sizes. Notably, no significant effects were observed for fat mass, body fat percentage, waist circumference, or functional performance. Although longer intervention durations (≥12 weeks) appeared more likely to produce significant improvements in fat-free mass, muscle mass, and muscle strength, this pattern may partly reflect the predominance of long-duration studies among those included. Moreover, participant characteristics (e.g., age, BMI), the timing of supplementation, caloric equivalence of study design, and exercise interventions may further modulate these adaptations. Given the preliminary nature of the evidence, furthermore, more high-quality, female-specific RCTs are needed to confirm these effects.

### 4.1 Body composition

Regarding body composition, our meta-analysis demonstrated that MIPS combined with exercise training significantly increased fat-free mass (0.45 kg) in adult women, although no statistically significant effects were observed for fat mass, body fat percentage, or waist circumference. These findings are partly consistent with previous meta-analyses, such as Bryan et al. (17), who reported significant improvements in fat-free mass (0.80 kg) with MIPS combined with RT in general adult populations. In contrast, Puente-Fernández et al. (22) observed no significant effects of MIPS on lean or fat-free mass (*g* = 0.044) in middle-aged and older adults. This discrepancy may be attributable to differences in age, sex distribution, and baseline health status across studies. Although their use of isoenergetic, non-protein comparators enhanced methodological rigor, the participants were primarily male and non-obese groups that may respond differently to supplementation. The discrepancies observed across studies may therefore reflect population-specific effects. Notably, our review is among the first to systematically investigate the effects of MIPS in exclusively female populations. For instance, both Bryan et al. (17) and Puente-Fernández et al. (22) primarily included male participants, limiting the generalizability of their findings to women and obscuring potential sex-specific responses. Given the well-established sex differences in muscle metabolism, hormonal regulation, and training adaptations, our findings provide critical insight into female-specific responses. The absence of improvements in fat mass and waist circumference, despite gains in fat-free mass, highlights the need for gender-tailored approaches in both research and clinical practice.

The observed increase in fat-free mass may be attributed to synergistic anabolic effects of MIPS components. High-quality proteins, particularly leucine-rich sources like whey, stimulate muscle protein synthesis via mTOR activation, which enhances translational efficiency and supports satellite cell activity in response to resistance training (18). Creatine enhances phosphocreatine resynthesis, supporting greater training volume and recovery (51), and enhancing muscle mass gain over RT alone (52). Meanwhile, vitamin D contributes to neuromuscular function and anabolic hormone regulation (20). Additional ingredients such as free leucine and polyunsaturated fatty acids may further support muscle accretion, particularly under suboptimal dietary conditions or in older adults (53). Collectively, these ingredients may act additively to amplify training adaptations beyond protein alone. Additionally, a recent review further supports the importance of optimizing protein intake strategies for women. It is proposed that daily protein needs for recreational and competitive premenopausal female athletes should range between 1.2–2.0 g/kg/day, with acute doses of 0.32–0.38 g/kg pre- or post-exercise shown to enhance recovery and training outcomes (54). Although sex hormones and menstrual cycle variations may influence protein metabolism (55), some current reviews suggest that men and women display broadly similar anabolic responses when protein is normalized to body or lean mass (56). As our meta-analysis did not directly assess mechanistic outcomes, more detailed physiological interpretations are beyond its scope.

Subgroup analyses suggest that improvements in fat-free mass are not consistent across all women but are moderated by key participant and intervention characteristics. For instance, greater gains were observed in older women, potentially due to increased sensitivity to anabolic stimuli, such as protein or creatine supplementation, against the backdrop of age-related declines in muscle mass and protein synthesis (57). Likewise, the observation that overweight, but not obese, participants demonstrated significant gains may reflect obesity-associated impairments in muscle protein synthesis or anabolic resistance, as reported in both clinical and mechanistic studies (58). These findings highlight the complexity of body composition as a moderating factor and suggest that MIPS efficacy may be diminished in individuals with more severe metabolic dysfunction. Importantly, caloric equivalence significantly moderated outcomes. Non-isocaloric RCTs showed clear gains in fat-free mass (+0.49 kg, *p* = 0.001), while isocaloric trials showed no effect (−0.16 kg, *p* = 0.68). This suggests that additional energy intake provided by MIPS may have contributed to anabolic adaptations, either directly (e.g., increased substrate availability) or indirectly (e.g., supporting higher training volume or better recovery). These results imply that true supplementation effects may be masked in isocaloric designs, particularly in women with already marginal energy intakes.

Supplement timing also emerged as an important determinant (59), with greater effects observed when MIPS were consumed near training sessions compared to near dietary intake. This supports the “anabolic window” hypothesis, which posits that nutrient timing near RT enhances muscle protein accretion, though this remains a topic of debate (60–62). Our findings provide indirect support for this concept in women, a population historically underrepresented in exercise science (11). Finally, intervention duration proved to be a critical factor. Only studies lasting longer than 12 weeks demonstrated significant improvements in fat-free mass, suggesting that adequate exposure time is required to produce measurable physiological adaptations. This observation is consistent with established periodization models and underscores the importance of sustained interventions when aiming to improve body composition. Collectively, these findings emphasize the need to tailor MIPS interventions not only by sex but also by age, body composition, supplementation timing, and program duration to optimize outcomes in women.

Together, these findings highlight the need for tailored MIPS combined exercise training strategies in women on body composition, accounting not just for sex differences, but also age, BMI category, nutrient timing, caloric load, and program duration.

### 4.2 Muscle fitness

Regarding muscle fitness, our meta-analysis demonstrated that MIPS combined with exercise training resulted in significant improvements in muscle mass and cross-sectional area [*g* = 0.35 (0.05, 0.65)] and strength [*g* = 0.50 (0.06, 0.95)] in women, compared with control groups. These findings align partially with previous studies, such as Bryan et al. (17), who reported significant gains in 1RM lower body (4.22 kg) and 1RM upper body (2.56 kg) with MIPS combined with resistance training. However, their analysis was heavily male-dominated, limiting its applicability to women. Similarly, Puente-Fernández et al. (22) found no significant effects of MTN formulations on muscle strength (e.g., *g* = 0.046 for 1RM bench press; *g* = 0.025 and 0.106 for 1RM leg press and leg extension, respectively), with negligible between-group differences and minimal heterogeneity. In contrast, our female-focused analysis highlights potential sex-specific responses to MIPS, particularly in muscle hypertrophy and strength development. This aligns with emerging evidence suggesting that women may exhibit distinct adaptations to resistance training and nutrient timing due to hormonal profiles and muscle fiber composition. The observed gains in muscle mass and strength may be attributed to the complementary actions of key MIPS ingredients. Among these, creatine likely played a central role, as its supplementation elevates high-energy phosphate resynthesis in skeletal muscle, promoting greater work capacity during short-duration exercise and increasing intramuscular water content (19). Other components, such as free leucine, may enhance post-exercise muscle protein synthesis via mTOR activation (63), while polyunsaturated fatty acids might help preserve muscle mass by modulating inflammation and protein turnover (53), though their additive effects with resistance training require further clarification. While these pathways suggest additive or synergistic effects with resistance training, further studies are warranted to delineate their interactive roles in women.

Age was a significant moderator for strength outcomes. Young adults demonstrated more pronounced improvements (*g* = 0.71) compared to older adults (*g* = 0.41). This may be attributed to age-related anabolic resistance, slower neuromuscular adaptation, or differing hormonal responses in older women (64, 65). The age-specific disparity in muscle strength, while not muscle mass, implies that while older women may still accrue morphological benefits from MIPS, their capacity to translate these changes into functional force output may be comparatively limited. Meanwhile, these findings should be interpreted with caution, as only three studies included young participants and five to six studies involved older adults. The limited sample sizes preclude strong conclusions, and the observed age-related differences should be regarded as preliminary and constrained by insufficient evidence.

Interestingly, nutrient timing also moderated outcomes, though the pattern varied by endpoint. For muscle mass, greater gains were observed when supplementation was timed near dietary intake (*g* = 0.45) rather than training (*g* = 0.29). This may reflect improved total daily nutrient utilization or enhanced anabolic synergy with meals. Conversely, muscle strength was more responsive when MIPS were consumed near dietary intake (*g* = 0.76 vs. *g* = 0.44), possibly due to indirect effects on recovery, energy availability, or neuromuscular readiness rather than acute protein synthesis alone. These findings challenge the traditional emphasis on the “anabolic window” (60–62) and suggest that strategic integration of MIPS within the broader nutritional context may be more influential in women. Intervention duration was a consistent determinant of both muscle mass and strength gains. Specifically, studies lasting longer than 12 weeks produced greater improvements (muscle mass: *g* = 0.46; strength: *g* = 0.86) compared to shorter interventions (*g* = 0.07 and *g* = 0.27, respectively). This temporal dependency aligns with the known time course of muscle hypertrophy and neural adaptation, indicating that shorter trials may fail to capture meaningful changes in muscle phenotype or performance capacity. Moreover, these results reinforce the need for sustained and progressive resistance training protocols when evaluating the effects of MIPS.

Importantly, caloric equivalence emerged as a key moderator of both outcomes. Non-isocaloric RCTs showed significant gains in muscle mass (*g* = 0.46) and strength (*g* = 0.71), whereas isocaloric designs yielded smaller, non-significant effects (muscle mass: *g* = 0.18; strength: *g* = 0.32). These findings suggest that additional energy provided by MIPS may contribute directly to muscle accretion and strength enhancement, possibly by supporting energy-demanding processes like protein synthesis, improving recovery capacity, or facilitating greater training outputs. From a physiological standpoint, this supports the principle that muscle anabolism is not solely dependent on amino acid availability, but also on sufficient energy intake, a particularly relevant consideration for active women who may face relative energy deficits. However, these findings raise methodological concerns, as non-isocaloric designs may confound the true effects of MIPS with added energy intake. Future studies should prioritize isocaloric controls to isolate supplement-specific effects and, when unavoidable, clearly report and account for energy differences through subgroup or sensitivity analyses. In contrast, protein dosage did not significantly moderate effects on either muscle mass or strength, with no subgroup differences detected. This lack of statistical significance may stem from the limited number of studies reporting exact protein dosages, heterogeneity in formulations, or inadequate statistical power. It is also possible that a threshold dose sufficient to stimulate maximal MPS was already met across most interventions (38), making further increases redundant in terms of measurable outcomes. Nonetheless, future trials should systematically assess protein quantity and quality to determine optimal dosing strategies for women.

Finally, no significant improvements were observed in muscle functional performance [*g* = 0.06 (−0.37, 0.49)], indicating that gains in muscle size and strength do not necessarily translate into better real-world function, at least in the short term. This disconnect may be due to insensitive testing protocols, ceiling effects in healthy populations, or the need for more complex neuromotor training to elicit functional improvements. It is also possible that some of the exercise protocols employed in the included studies were of insufficient intensity, duration, or specificity to drive detectable changes in functional outcomes, particularly in populations without marked baseline impairments, and especially when the exercise modalities themselves were not highly functional in nature. Future studies should incorporate ecologically valid and sex-sensitive functional metrics to better capture the applied value of MIPS interventions in diverse female populations.

### 4.3 Practical implication

The findings of this meta-analysis support several practical recommendations for implementing MIPS alongside exercise training in women. Most interventions utilized protein-based MIPS, often containing whey or casein with carbohydrates and occasionally including vitamin D, calcium, leucine, or creatine, typically providing 10 g/day (≈0.15 g/kg/day) to 45 g/day (≈0.6 g/kg/day) of protein and 5 to 31 grams of carbohydrates per serving. These supplements were generally consumed post-exercise or with meals, underscoring the importance of timing intake near training to maximize anabolic responses. Resistance training was the primary exercise modality across studies and should be prioritized in practice. Based on the reviewed protocols, programs should last at least 12 weeks with a frequency of three sessions per week. Training intensity is best progressed from moderate to higher loads, approximately 60 to 85% of one-repetition maximum, to continually stimulate muscular adaptation. Exercises should emphasize multi-joint movements that engage large muscle groups, such as squats, leg presses, deadlifts, chest presses, and rows, to promote comprehensive improvements in muscle mass and strength. Importantly, recommendations should be individualized. Older adults or individuals with limited training experience may benefit from starting with lighter loads and higher repetitions, gradually increasing intensity and reducing repetitions to ensure safe, progressive overload. Baseline nutritional status should also be considered, as women with suboptimal dietary protein intake may require higher relative supplementation. Recent evidence further suggests that premenopausal female athletes may require distinct protein dosing strategies to optimize muscle protein synthesis (54). While some interventions incorporated aerobic or interval training, primary benefits were observed for lean mass and strength. Therefore, additional aerobic volume or neuromotor exercises may be included when targeting fat reduction or functional outcomes. Moreover, incorporating balance and coordination training can further enhance neuromuscular control and daily functional capacity, particularly in older populations. Overall, these findings highlight the value of combining protein-rich MIPS with a structured, progressively overloaded resistance training program tailored in duration, frequency, and intensity to individual characteristics such as age, baseline nutrition, and training status. Practitioners should align program goals with realistic expectations and apply these insights to develop personalized interventions that effectively improve muscle mass, strength, and overall musculoskeletal health in women.

### 4.4 Future direction

Future research should address several critical gaps identified in this meta-analysis. First, to enhance the generalizability of findings, studies should move beyond healthy, non-clinical populations to include women with chronic conditions such as sarcopenia, metabolic syndrome, and cardiovascular disease. Evaluating MIPS efficacy in these higher-risk groups would provide valuable insights into its potential therapeutic and preventive applications among those most likely to benefit.

Second, given that most existing trials have employed moderate-intensity resistance training, future research should incorporate a wider array of exercise modalities. In particular, studies examining aerobic training, combined aerobic and resistance (concurrent) exercise, and high-intensity interval training (HIIT) alongside MIPS are warranted. Such investigations would help determine whether different exercise stimuli modulate the effects of MIPS on muscle morphology, fat distribution, and functional performance.

Third, there is a clear need to standardize and rigorously evaluate MIPS formulations. The substantial variability in ingredient composition and dosing across studies complicates attributing observed benefits to specific nutrients or combinations. Future trials should consider factorial or multi-arm designs to disentangle the independent and interactive effects of key MIPS components such as creatine, protein, and caffeine, thereby informing evidence-based supplementation strategies. In this context, dose–response relationships, particularly for protein, remain poorly understood in women. Future research should examine individualized protein dosing relative to body weight or lean mass, and clarify whether additional protein above the threshold confers added benefit, especially when combined with other MIPS components.

Additionally, caloric equivalence remains a critical but under-addressed methodological issue. Future studies should employ rigorously controlled isocaloric designs to isolate supplement-specific effects and avoid confounding from energy imbalance. Where non-isocaloric comparisons are necessary, energy differentials should be explicitly reported and statistically accounted for, possibly through stratified or sensitivity analyses. Such methodological refinements will enhance internal validity and comparability across trials.

Moreover, meticulous control and transparent reporting of exercise intervention characteristics are essential. Many prior studies have lacked detailed descriptions of adherence, progression, and volume-load metrics, which are critical for interpreting how MIPS interacts with training adaptations. Standardized reporting would greatly improve reproducibility and facilitate more precise synthesis in future meta-analyses. Future investigations should also explore inter-individual variability in responsiveness to combined MIPS and exercise interventions, including the prevalence and characteristics of responders and non-responders. Identifying factors that contribute to such heterogeneity, including baseline nutritional status, hormonal profiles, genetic polymorphisms, or microbiome composition, would be instrumental in advancing personalized strategies. In particular, future research should investigate sex-specific determinants of responsiveness. Clarifying these sex-based differences will be essential for developing tailored supplementation and training guidelines that more effectively optimize outcomes in female populations.

Finally, longer intervention durations and extended follow-up periods are needed to evaluate the sustainability of MIPS-related adaptations. While short-term improvements in muscle mass and strength are promising, it remains unclear whether these gains persist over time or translate into meaningful reductions in disability and chronic disease risk. Incorporating mechanistic assessments, such as muscle biopsies or molecular profiling, could further elucidate the biological pathways involved and refine our understanding of individual differences in treatment response.

Collectively, these recommendations underscore the need for large-scale, well-powered randomized controlled trials that incorporate diverse populations, standardized interventions, isocaloric controls, dose–response analyses, and rigorous methodologies to advance our understanding of how MIPS can optimize health and functional outcomes in women.

### 4.5 Limitation

Several limitations of this meta-analysis warrant consideration. First, the included studies were limited to peer-reviewed publications written in English, which introduces potential language and publication bias. Second, the number of eligible studies was relatively small (n = 9), which limits the statistical power, particularly for certain subgroup or outcome analyses (e.g., waist circumference, functional performance). Third, the participant population consisted exclusively of healthy, non-clinical women. The absence of trials involving women with chronic conditions such as sarcopenia, metabolic syndrome, or cardiovascular disease restricts the generalizability of findings to broader or more clinically relevant populations. Fourth, while the MIPS formulations varied across studies, the majority of exercise interventions were based on moderate-intensity resistance training. There was a marked lack of studies involving aerobic exercise or combined training protocols, limiting conclusions regarding the interaction between MIPS and different modes of exercise. Fifth, there was substantial heterogeneity in MIPS formulations, including differences in the presence, type, and dosage of key components such as creatine, protein, caffeine, and other nutrients. In particular, the source and quality of protein (e.g., whey, casein, plant-based) varied across studies, as did the protein dosing protocols, precluding any definitive dose–response analysis. Sixth, the absence of isocaloric comparator designs in some studies raises concerns about energy mismatch, which may confound supplement-specific effects by attributing benefits to added caloric intake rather than MIPS ingredients alone. Seventh, the duration and periodization of interventions were inconsistent, with some studies not reporting adherence, progression, or volume-load details, key factors in interpreting training-related adaptations. Lastly, although subgroup analyses provided useful insights, they remain exploratory due to small sample sizes within strata and should be interpreted cautiously. Future large-scale, well-powered randomized controlled trials are needed to validate these observations, especially across diverse training modalities and clinical populations.

## 5 Conclusion

This current meta-analysis evidence indicates that combining MIPS and exercise training may provide modest benefits for fat-free mass, muscle mass, and muscle strength, while not conferring additional benefits for fat mass, body fat percentage, waist circumference, or functional performance. Subgroup analyses further reveal that these benefits are moderated by age, BMI, supplementation timing, and intervention duration, suggesting that individual characteristics and program design might influence outcomes. However, these findings should be interpreted with caution, given the small number of included studies and relatively limited sample sizes. Given the underrepresentation of women in sports nutrition research and the heterogeneity in supplement composition and training protocols, future high-quality trials in diverse female populations, including clinical groups, are urgently needed. These results underscore the importance of tailoring MIPS interventions to female-specific physiological contexts to optimize health and performance outcomes.

## Data Availability

The original contributions presented in the study are included in the article/Supplementary material, further inquiries can be directed to the corresponding author.

## Footnotes

^1^ https://osf.io/
