# Supplementary material for: Effects of multi-ingredient protein supplementation combined with exercise intervention on body composition and muscle fitness in healthy women: a systematic review with multilevel meta-analysis
Source: Front Nutr. 2025 Nov 3;12:1678433. doi: 10.3389/fnut.2025.1678433 (PMC12622227; doi:10.3389/fnut.2025.1678433)
Supplement: Supplementary file 2 [file Supplementary_file_2.docx]

Appendix B. Statistical Power Sunset Chart.


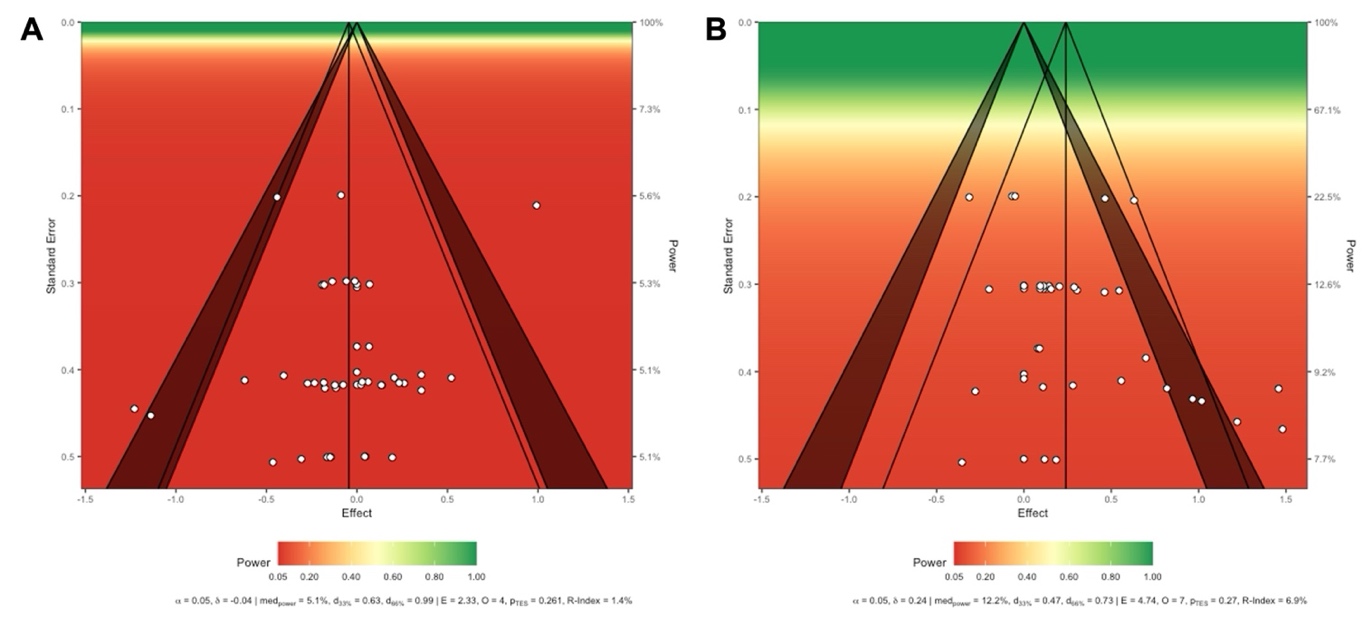


**NOTE:** Sunset funnel plots illustrating statistical power and the potential for publication bias across included studies. Panel (A) depicts the power plot for body composition outcomes, while panel (B) shows the power plot for muscle fitness outcomes. The color gradient represents statistical power, ranging from red (lower power) to green (higher power). Each white dot corresponds to an individual study, plotted according to its effect size and standard error.
