# Supplementary material for: Effects of multi-ingredient protein supplementation combined with exercise intervention on body composition and muscle fitness in healthy women: a systematic review with multilevel meta-analysis
Source: Front Nutr. 2025 Nov 3;12:1678433. doi: 10.3389/fnut.2025.1678433 (PMC12622227; doi:10.3389/fnut.2025.1678433)
Supplement: Supplementary file 3 [file Supplementary_file_3.docx]

Appendix C. Forest plot of combined body composition.


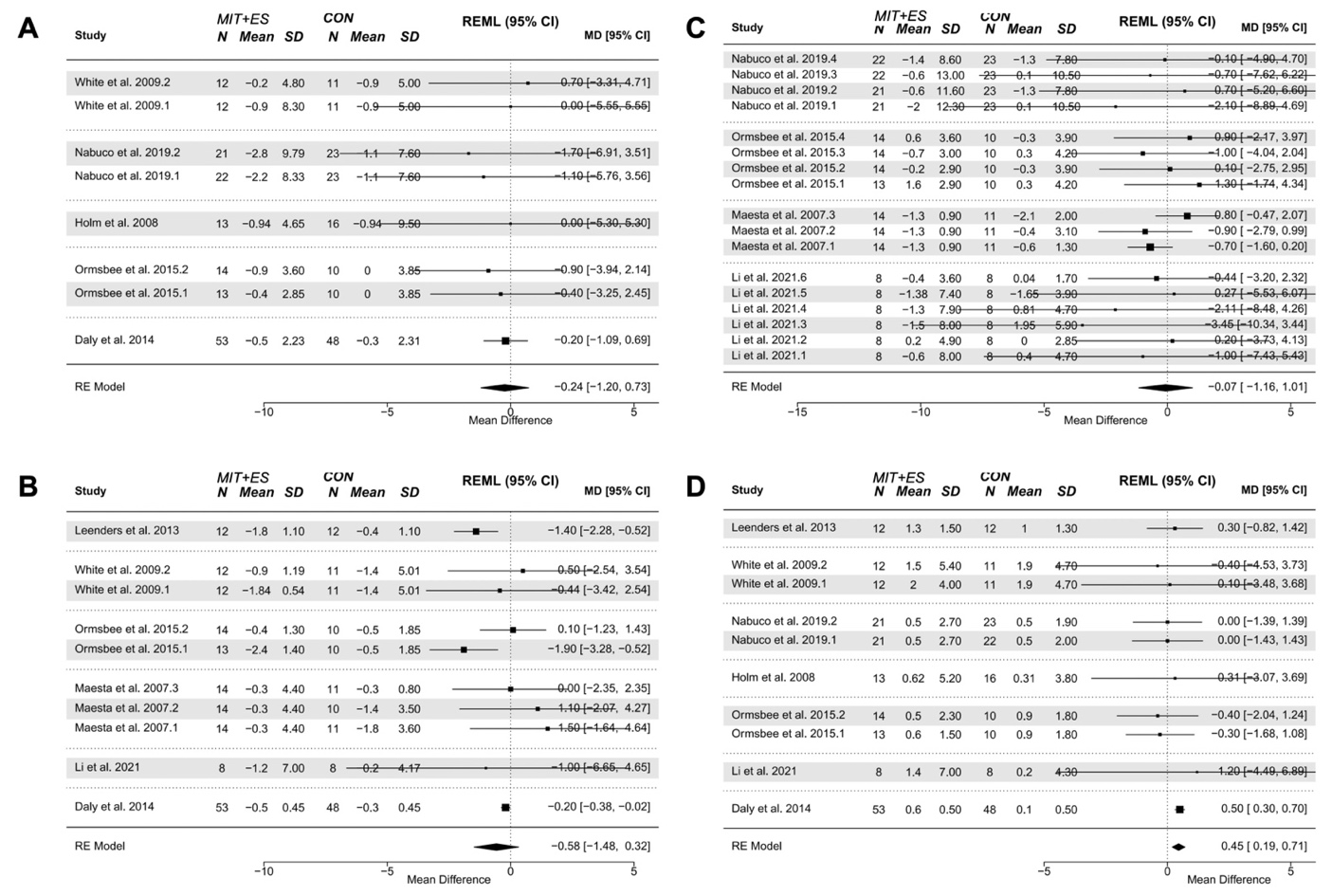


**NOTE:** Forest plots summarizing the effects of multi-ingredient protein supplementation combined with exercise training compared to control conditions on various body composition outcomes in women. Panel (A) shows the results for fat mass, panel (B) for body fat percentage, panel (C) for waist and hip circumference measures, and panel (D) for fat-free mass.
