# Supplementary material for: Effects of multi-ingredient protein supplementation combined with exercise intervention on body composition and muscle fitness in healthy women: a systematic review with multilevel meta-analysis
Source: Front Nutr. 2025 Nov 3;12:1678433. doi: 10.3389/fnut.2025.1678433 (PMC12622227; doi:10.3389/fnut.2025.1678433)
Supplement: Supplementary file 4 [file Supplementary_file_4.docx]

Appendix D. Forest plot of muscle fitness.


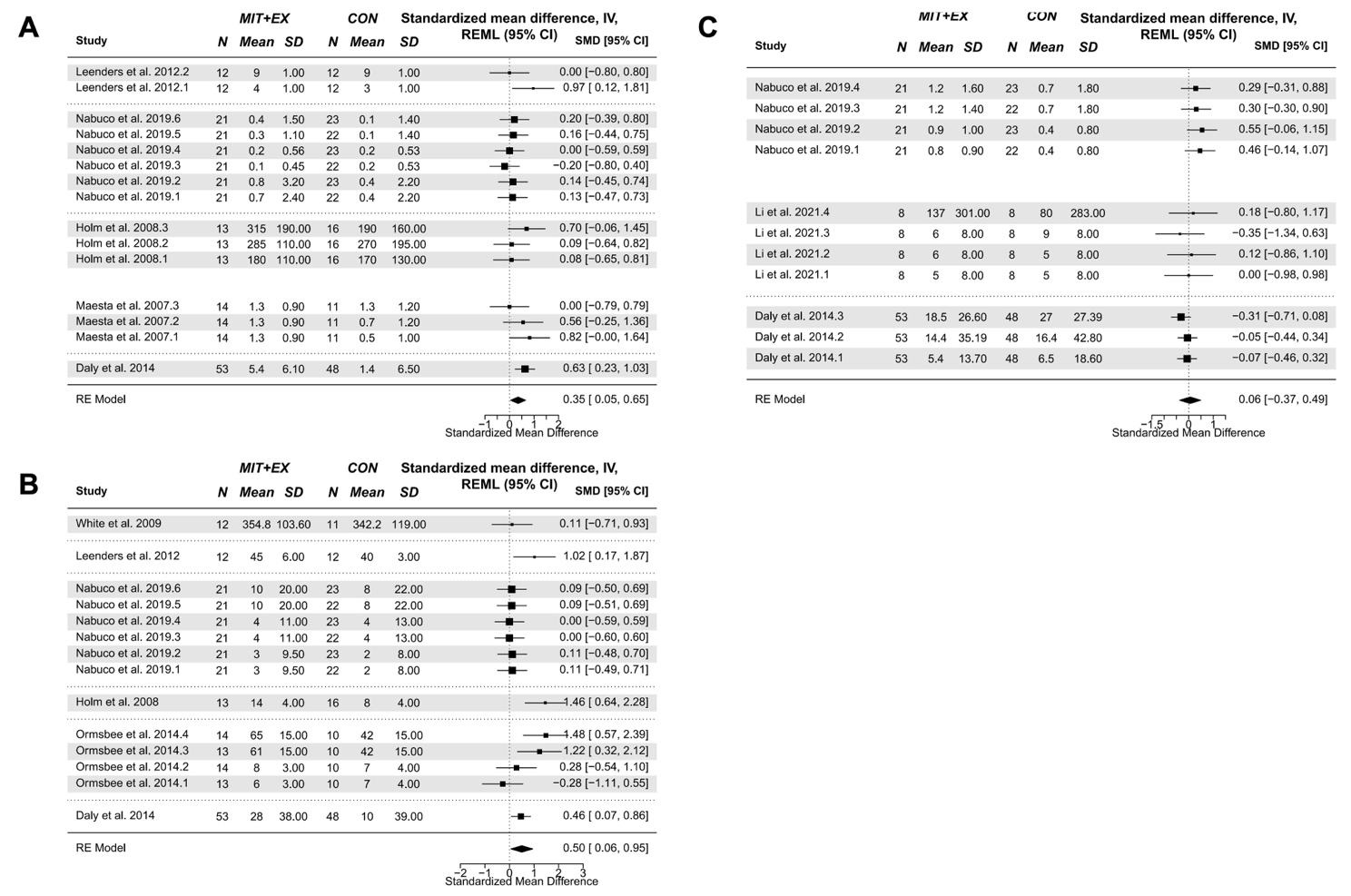


**NOTE:** Forest plots summarizing the effects of multi-ingredient protein supplementation combined with exercise training compared to control conditions on muscle-related outcomes in women. Panel (A) illustrates results for muscle hypertrophy parameters, panel (B) presents outcomes related to muscle strength, and panel (C) shows findings for muscle functional performance.
