# Supplementary material for: Effects of multi-ingredient protein supplementation combined with exercise intervention on body composition and muscle fitness in healthy women: a systematic review with multilevel meta-analysis
Source: Front Nutr. 2025 Nov 3;12:1678433. doi: 10.3389/fnut.2025.1678433 (PMC12622227; doi:10.3389/fnut.2025.1678433)
Supplement: Supplementary file 6 [file Supplementary_file_6.docx]

Appendix F. Sensitivity analysis regarding muscle fitness.


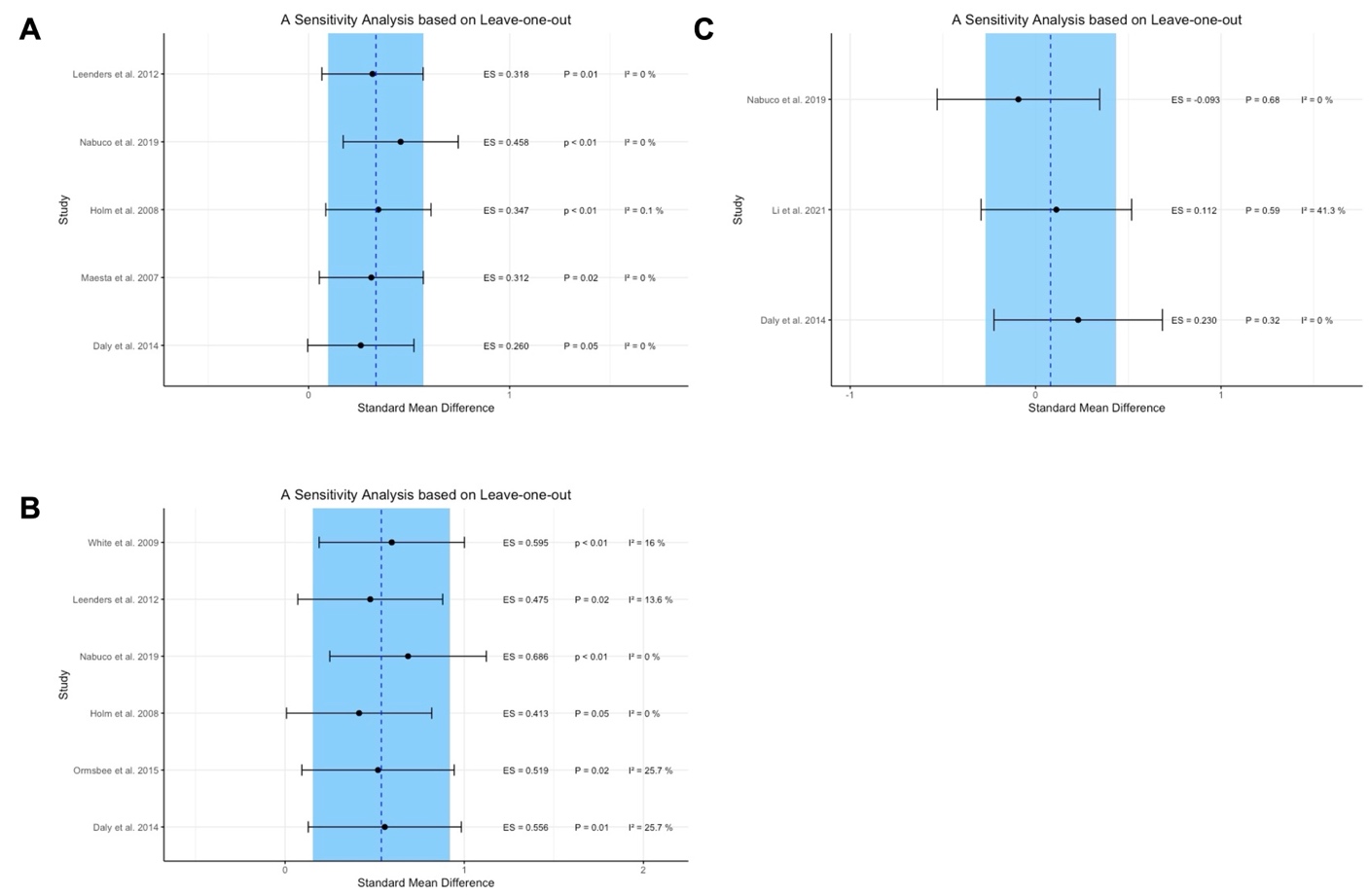


**NOTE:** Sensitivity plots summarizing the effects of multi-ingredient protein supplementation combined with exercise training compared to control conditions on various body composition outcomes in women. Panel (A) illustrates results for muscle hypertrophy parameters, panel (B) presents outcomes related to muscle strength, and panel (C) shows findings for muscle functional performance.
