# Supplementary material for: Effects of multi-ingredient protein supplementation combined with exercise intervention on body composition and muscle fitness in healthy women: a systematic review with multilevel meta-analysis
Source: Front Nutr. 2025 Nov 3;12:1678433. doi: 10.3389/fnut.2025.1678433 (PMC12622227; doi:10.3389/fnut.2025.1678433)
Supplement: Supplementary file 7 [file Table_1.docx]

**Supplementary  Table 1.** The characteristics for the studies included.

|  |  |  |  |  | **Training Protocols** | | | | |  |
| --- | --- | --- | --- | --- | --- | --- | --- | --- | --- | --- |
| **First author, year** | **Country** | **Design** | **Funding** | **Participants** | **Multi-Ingredient Protein Supplements Combined with Exercise Training group** | **Exercise Training group** | **Fre** | **Duration** | **Wk** | **PEDro** |
| Daly, 2014 | Australia | RCT | Yes | N: MIPS+RT (53); RT (48)  Age: MIPS+RT (72.1); RT (73.6)  BMI: MIPS+RT (27.76); RT (27.66)  Condition: Healthy | Supplement type: Lean red meat  Supplement details: Protein and vitamin D3 capsulest  Supplement timing: After training (160g/day cooked)  Doses per day: 2  Exercise type: RT  Other characters: 3 groups 8-12 repetitions | Supplement type: Low - protein diet  Supplement details: Low-protein diet and vitamin D3 capsules  Supplement timing: After training (25-35g carbohydrate/day)  Doses per day: 2  Exercise type: RT | 2 | 45-60 min | 16 | 7 |
| Li, 2021 | Taiwan | RCT | No | N: MIPS+AT (8); AT (8)  Age: MIPS+AT (38); AT (34)  BMI: MIPS+AT (21.11); AT (23.55)  Condition: Healthy | Supplement type: Protein supplement  Supplement details: 72 kcal, 11.6 g protein, 0.4 g fat, 5.5 g carbohydrate, 87 mg sodium  Supplement timing: After training  Doses per day: 1  Exercise type: AT  Other characters: 40-65% HRR | Supplement type: Equal volume of water  Supplement details: Equal volume of water  Supplement timing: After training  Doses per day: 1  Exercise type: AT | 2 | 60 min | 8 | 6 |
| Maesta, 2007 | Brazil | RCT | Yes | N: MIPS+RT (14); RT (11); MIPS (10); CON (11)  Age: MIPS+RT (57.6); RT (60.7); MIPS (61.3); CON (57.9)  BMI: MIPS+RT (27.8); RT: (27.7); MIPS: (27.2); CON: (26.6)  Condition: Healthy | Supplement type: Protein - milk mixed supplement  Supplement details: 0.31 g lipid, 12.2 g protein, 10 g carbohydrate, 0.7 g fiber, and 92 kcal  Supplement timing: Breakfast or lunch  Doses per day: 1  Exercise type: RT  Other characters: 60-80% 1-RM; MIPS group did not receive training. | Supplement type: Isocaloric maltodextrin  Supplement details: Isocaloric maltodextrin  Supplement timing: Breakfast or lunch  Doses per day: 1  Exercise type: RT  Other characters: Con group did not receive training and supply. | 3 | 40-50 min | 16 | 6 |
| Ormsbee,2015 | USA | RCT | Yes | N: MIPS+CT-a: (13); MIPS+CT-b (14); RT (10)  Age: MIPS+CT-a (27.7); MIPS+CT-b (29.3); RT (30)  BMI: MIPS+CT-a (33.1); MIPS+CT-b (34.4); RT (36.5)  Condition: Healthy | Supplement type: Whey protein supplement (CTa); Casein mixed supplement (CTb)  Supplement details: 50% whey protein isolate and concentrate mixture, 4 g carbohydrate, 1.5 g fat (CTa); 30 g micellar casein, 3 g carbohydrate, 0.5 g fat (CTb)  Supplement timing: After dinner  Doses per day: 1  Exercise type: RT + HIIT  Other characters: 70%-85%1RM | Supplement type: Isocaloric carbohydrate  Supplement details: Isocaloric carbohydrate  Supplement timing: After dinner  Doses per day: 1  Exercise type: RT + HIIT | 3 | 60 min | 4 | 6 |
| Holm,2008 | Denmark | RCT | No | N: MIPS+RT (13); RT (16)  Age: MIPS+RT (55); RT (55)  BMI: MIPS+RT (24); RT (27)  Condition: Healthy | Supplement type: Whey protein  Supplement details: 10 g protein (whey protein), 31 g carbohydrate, 1 g fat, 5.0 g vitamin D, and 250 mg calcium  Supplement timing: After training  Doses per day: 1  Exercise type: RT  Other characters: RT includes high foot position and low foot position. | Supplement type: Placebo  Supplement details: 6 g carbohydrate and 12 mg calcium  Supplement timing: After training  Doses per day: 1  Exercise type: RT  Other characters: RT includes high foot position and low foot position. | 3 | NR | 24 | 6 |
| Nabuco, 2018 | Brazil | RCT | Yes | N: MIPS+RT-1 (22); MIPS+RT-2 (21); RT (23)  Age: MIPS+RT-1 (67.5); MIPS+RT-2 (66.2); RT (66.5)  BMI: MIPS+RT-1 (26.4); MIPS+RT-2 (25.3); RT (23.8)  Condition: Healthy | Supplement type: Whey protein beverage (RT1); Whey protein beverage (RT2)  Supplement details: 27.1 g protein, 5.2 g carbohydrate, and 0.2 g fat (RT1); 27.1 g protein, 5.2 g carbohydrate, and 0.2 g fat (RT2)  Supplement timing: Whey protein beverage before training; placebo after training (RT1); Placebo before training; whey protein beverage after training (RT2)  Doses per day: 1  Exercise type: RT  Other characters: 8-12RM | Supplement type: Placebo  Supplement details: 0.3 g protein and 33.3 g carbohydrate  Supplement timing: Before and after training  Doses per day: 2  Exercise type: RT  Other characters: 8-12RM | 3 | NR | 12 | 6 |
| Nabuco, 2019 | Brazil | RCT | Yes | N: MIPS+RT-1 (22); MIPS+RT-2 (21); RT (23)  Age: MIPS+RT-1 (67.5); MIPS+RT-2 (66.2); RT (66.5)  BMI: MIPS+RT-1 (26.4); MIPS+RT-2 (25.3); RT (23.8)  Condition: Healthy | Supplement type: Whey protein beverage (RT1); Whey protein beverage (RT2)  Supplement details: 27.1 g protein, 5.2 g carbohydrate, and 0.2 g fat (RT1); 27.1 g protein, 5.2 g carbohydrate, and 0.2 g fat (RT2)  Supplement timing: Whey protein beverage before training; placebo after training (RT1); Placebo before training; whey protein beverage after training (RT2)  Doses per day: 1  Exercise type: RT  Other characters: 8-12RM | Supplement type: Placebo  Supplement details: 0.3 g protein and 33.3 g carbohydrate  Supplement timing: Before and after training  Doses per day: 2  Exercise type: RT  Other characters: 8-12RM | 3 | NR | 12 | 6 |
| Leenders, 2013 | Netherlands | RCT | No | N: MIPS+RT-1(female) (12)  RT-1 (12)  Age: MIPS+RT-1(female) (72)  RT-1 (69)  BMI: MIPS+RT-1(female) (24.2)  RT-1 (25)  Condition: Healthy | Supplement type: Protein beverage  Supplement details: 15 g protein (milk protein concentrate (MPC 80); DMV International, Delhi, NY), 0.5 g fat, 7.13 g lactose, and 0.42 g calcium  Supplement timing: Before breakfast  Doses per day: 1  Exercise type: RT  Other characters: 60%-80%1RM | Supplement type: Placebo  Supplement details: 7.13 g lactose and 0.42 g calcium  Supplement timing: Before breakfast  Doses per day: 1  Exercise type: RT  Other characters: 60%-80%1RM | 3 | NR | 24 | 6 |
| Whites, 2009 | USA | RCT | Yes | N: MIPS+RT (12)  RT (11)  Age: MIPS+RT (18.8)  RT (19.3)  BMI: MIPS+RT: NR  RT: NR  Condition: Healthy | Supplement type: Post - exercise supplement group of dairy calcium plus carbohydrate and protein  Supplement details: Accel Gel (90 calories, 20 g carbohydrate, 5 g protein; PacificHealth Laboratories, Inc., Matawan, NJ)  Supplement timing: After training  Doses per day: 1  Exercise type: RT  Other characters: To ensure appropriate progression, exercise prescription is based on the Daily Adjustable Progressive Resistance Exercise (DAPRE) system. | Supplement type: Post - exercise supplement group of dairy calcium plus carbohydrate only  Supplement details: 7.13 g lactose and 0.42 g calcium  Supplement timing: Before breakfast  Doses per day: 1  Exercise type: RT  Other characters: To ensure appropriate progression, exercise prescription is based on the Daily Adjustable Progressive Resistance Exercise (DAPRE) system. | 3 | NR | 8 | 5 |

***Note*: *N****,* sample size; ***Fre****,* training frequency (sessions/week); ***Wk****, t*raining intervention weeks; ***RCT****,* randomised controlled trial; ***RT****,* resistance training; ***AT****,* aerobic training; ***CT****,* resistance training + high intensity interval training; ***MIPS****, m*ulti-ingredient protein supplements; ***NR***, not report.
